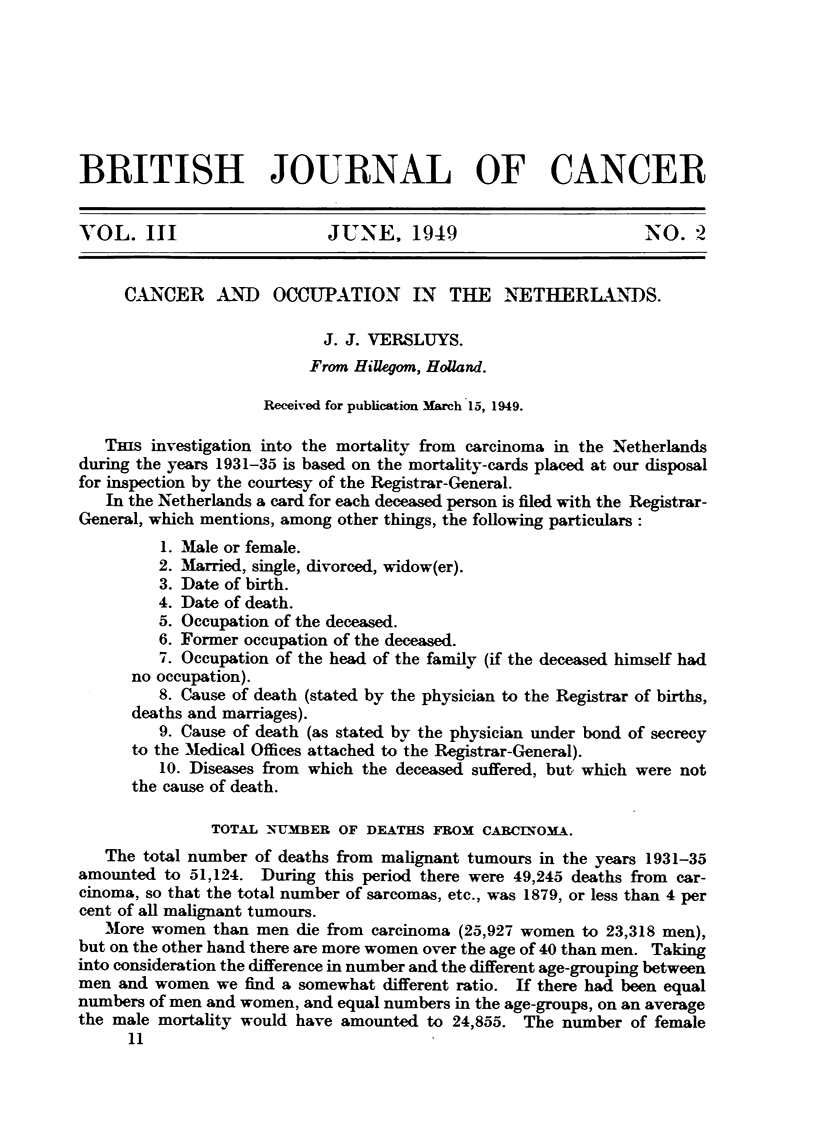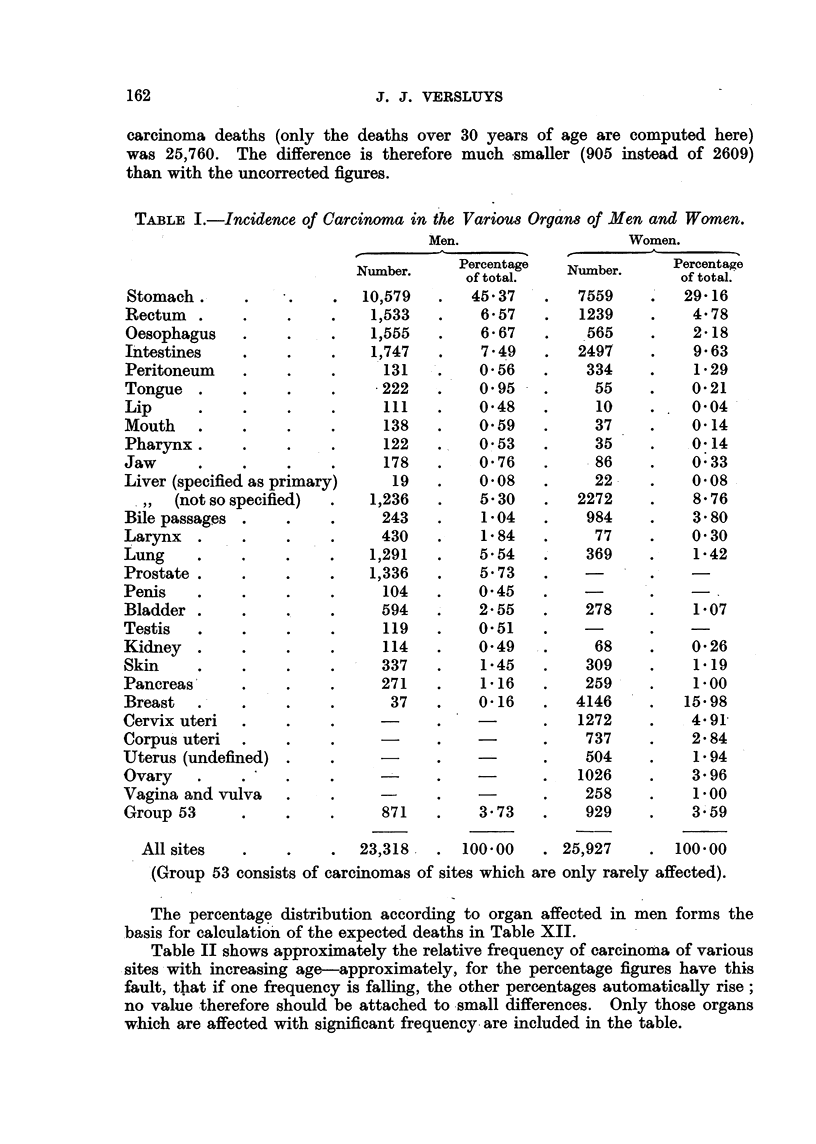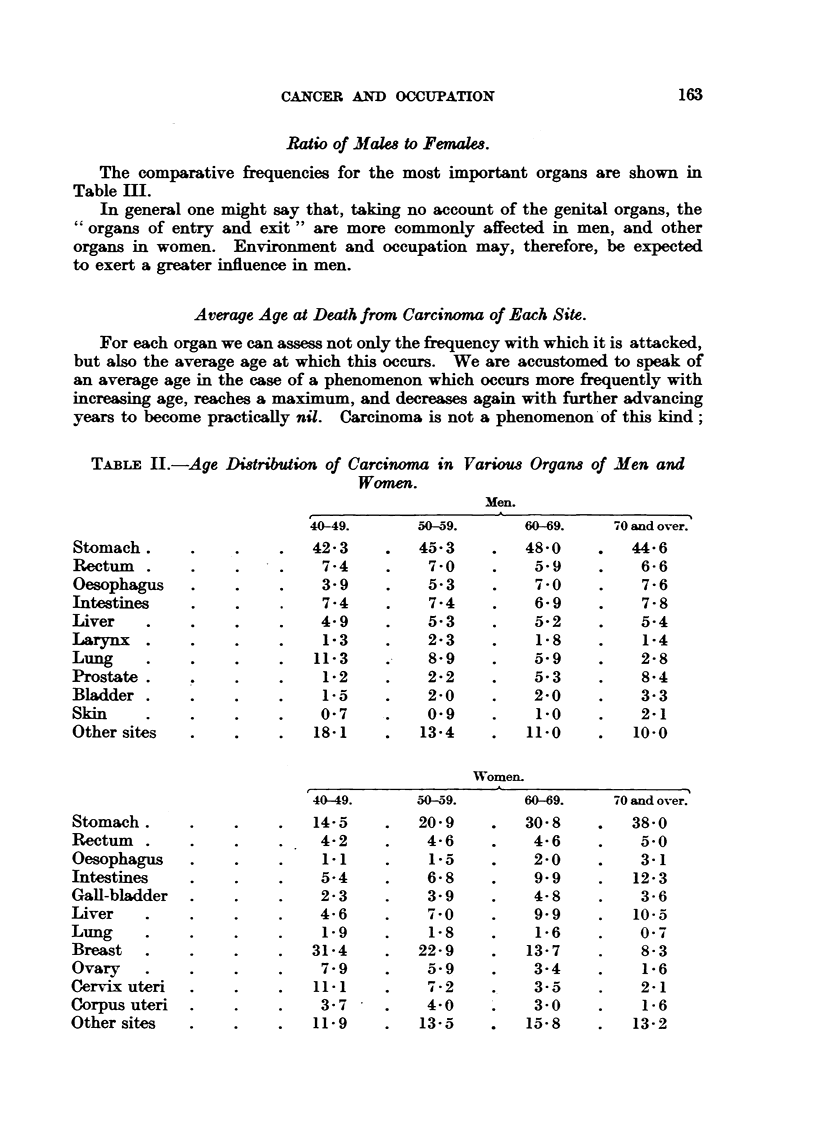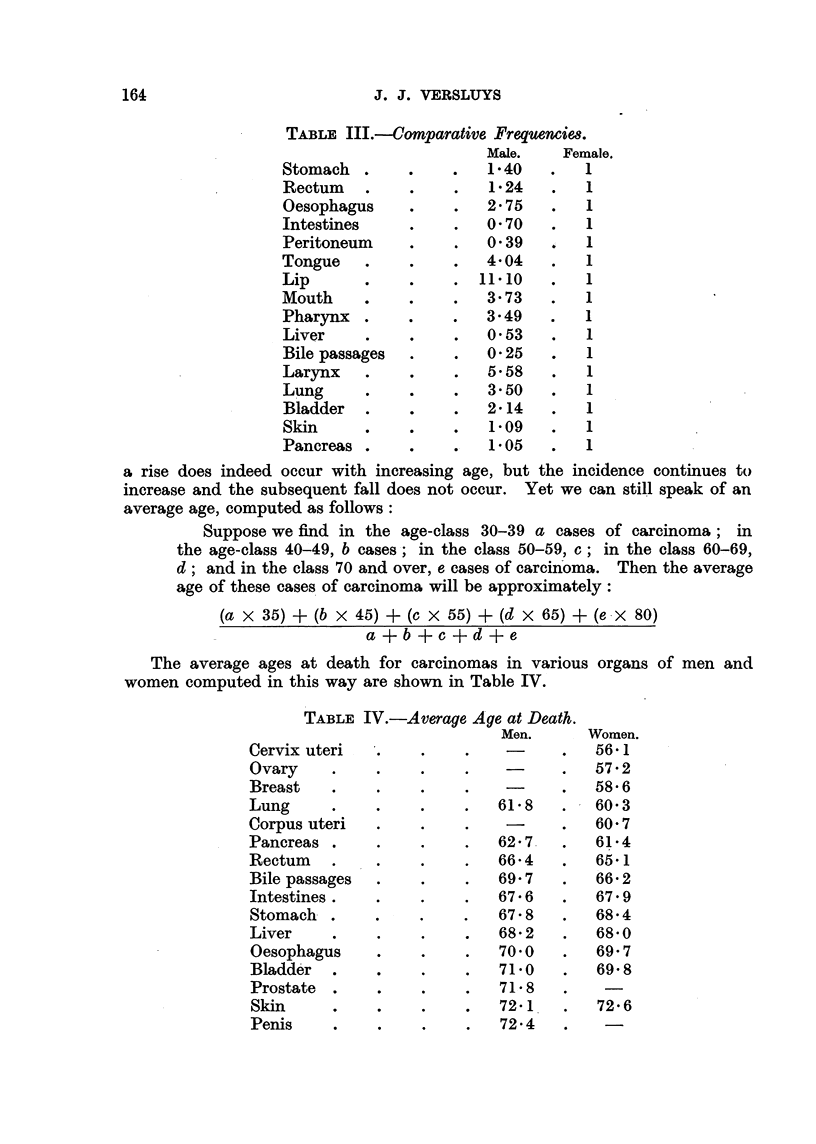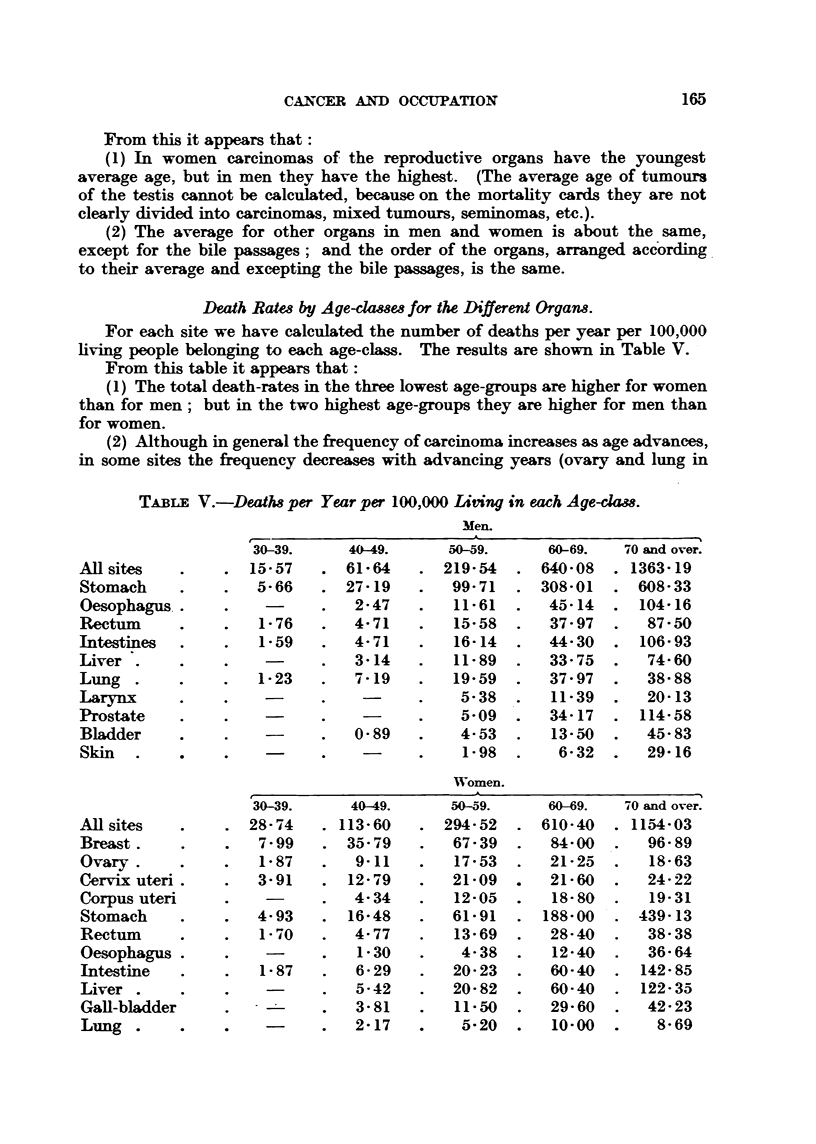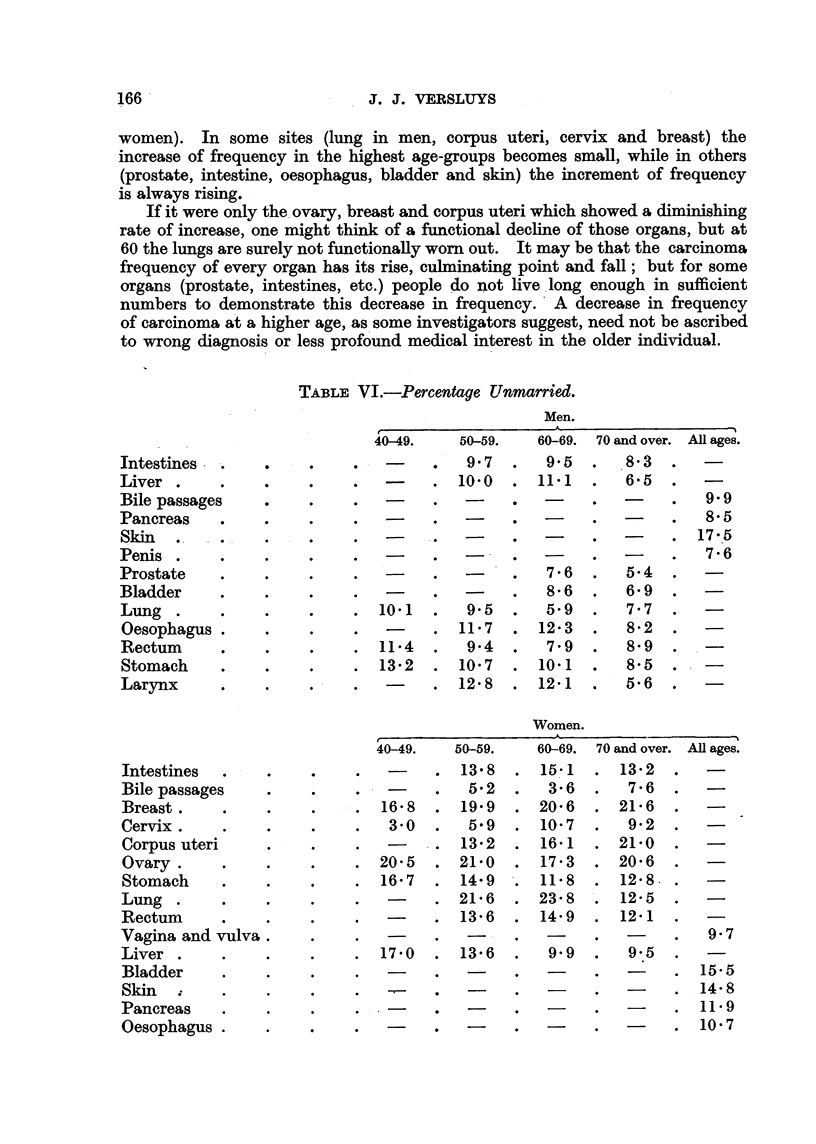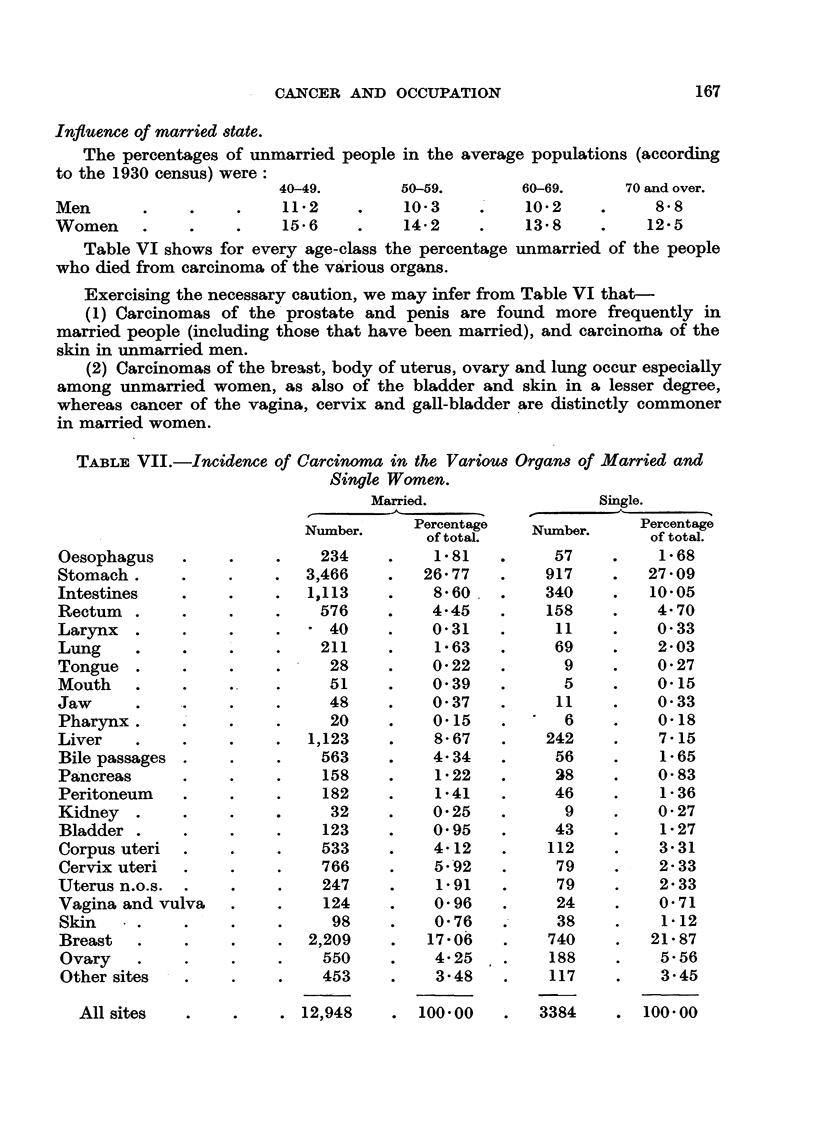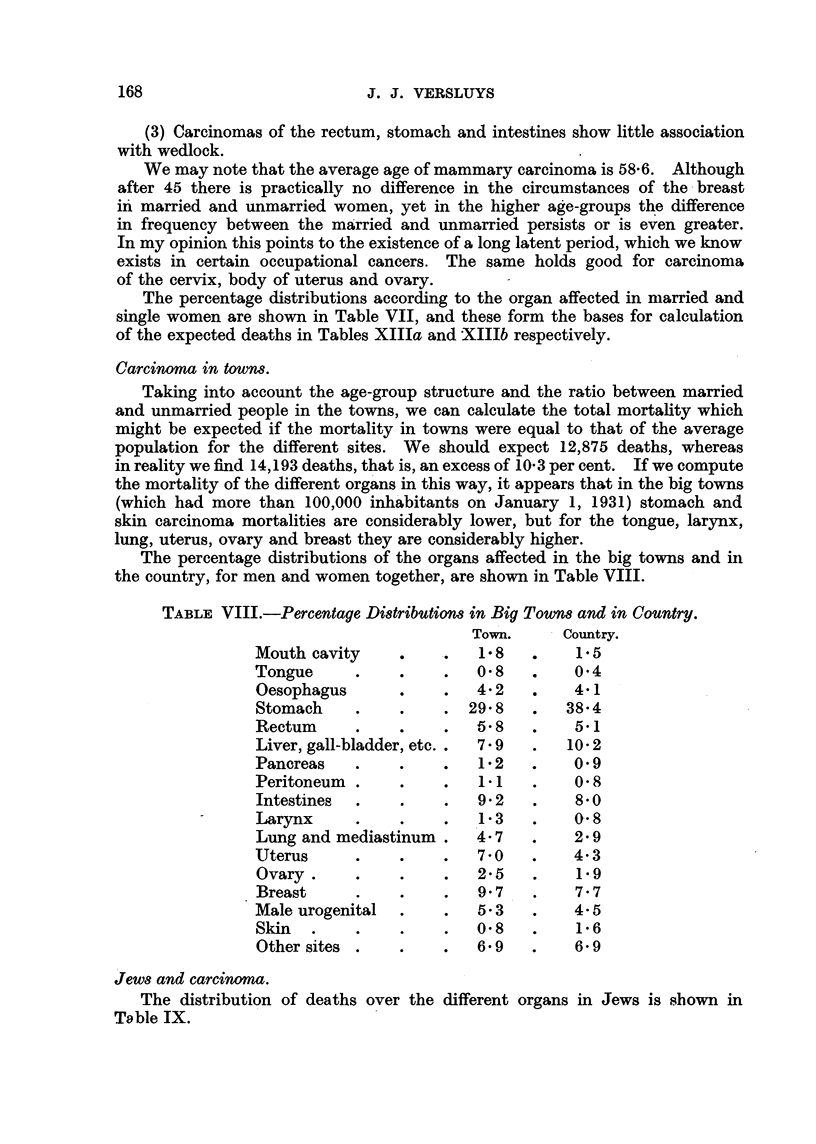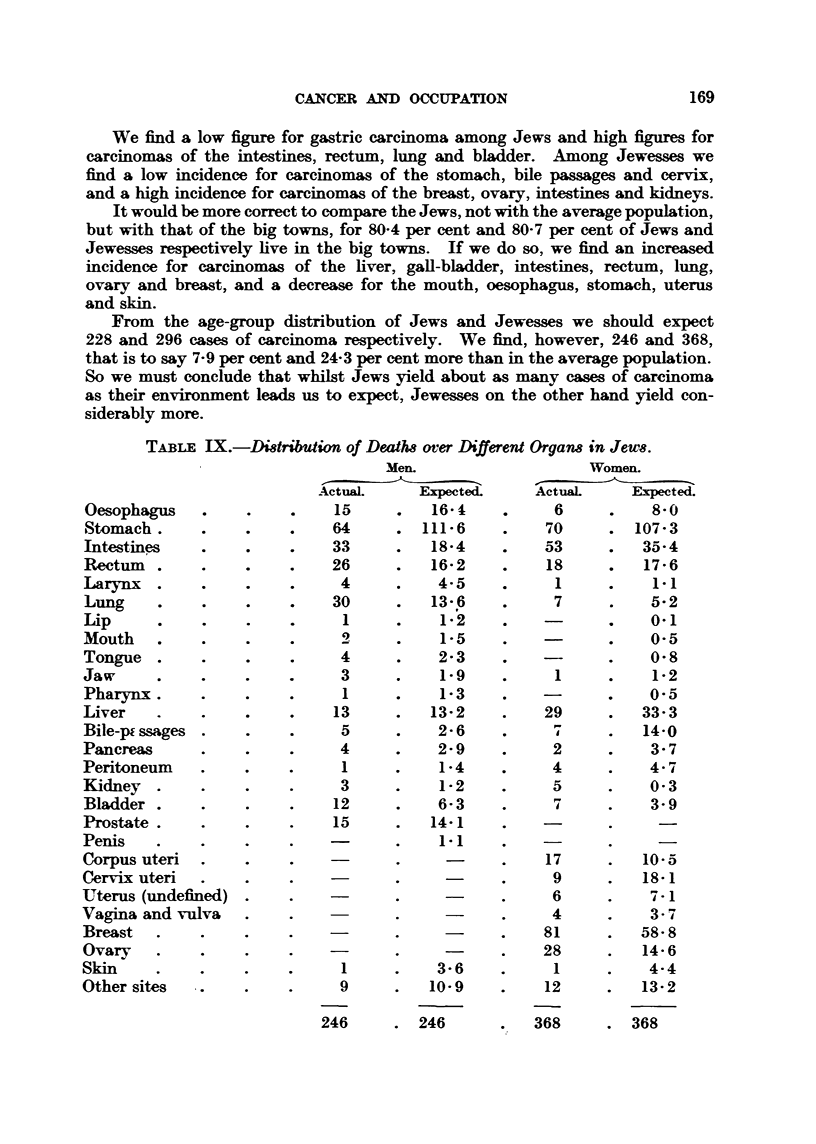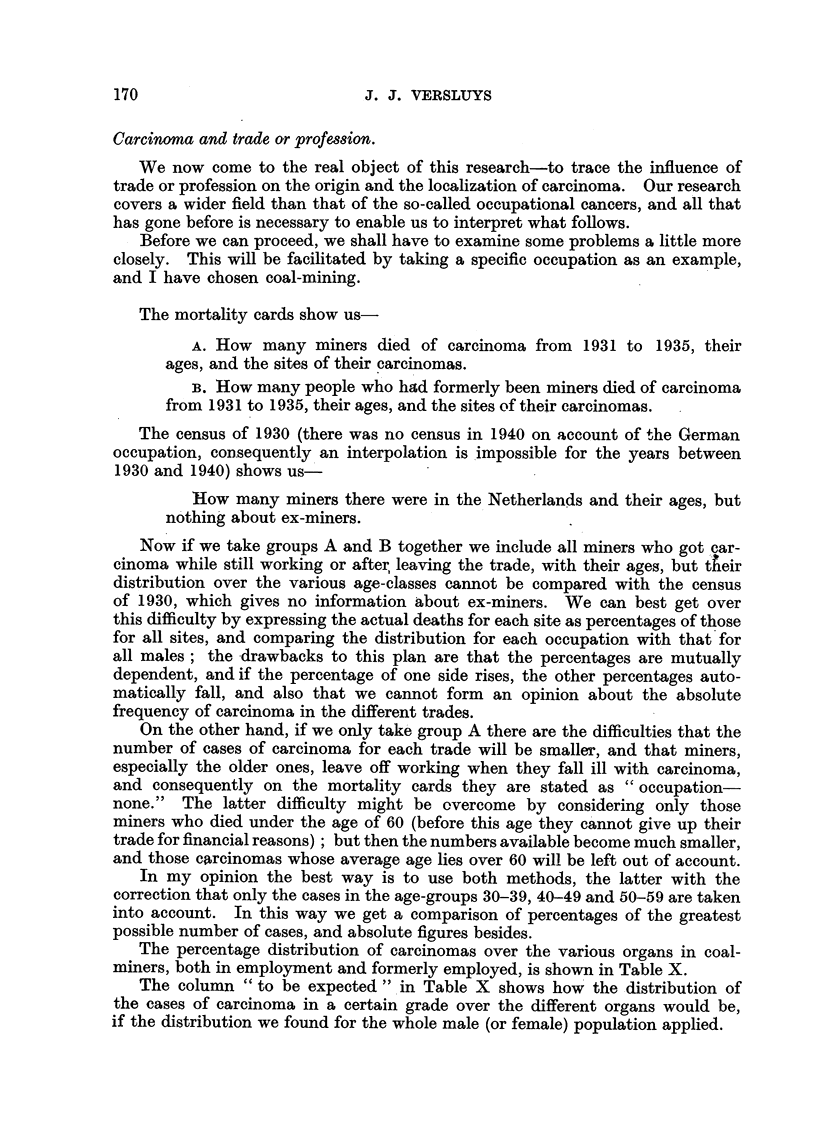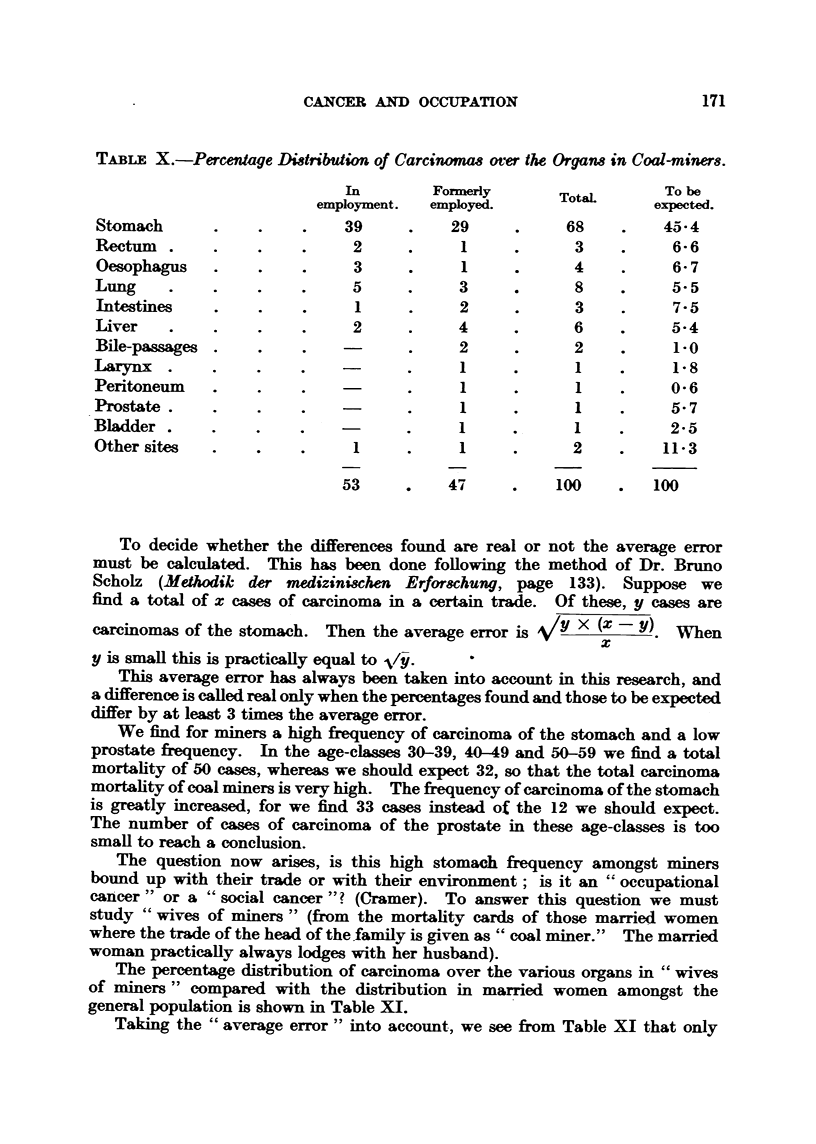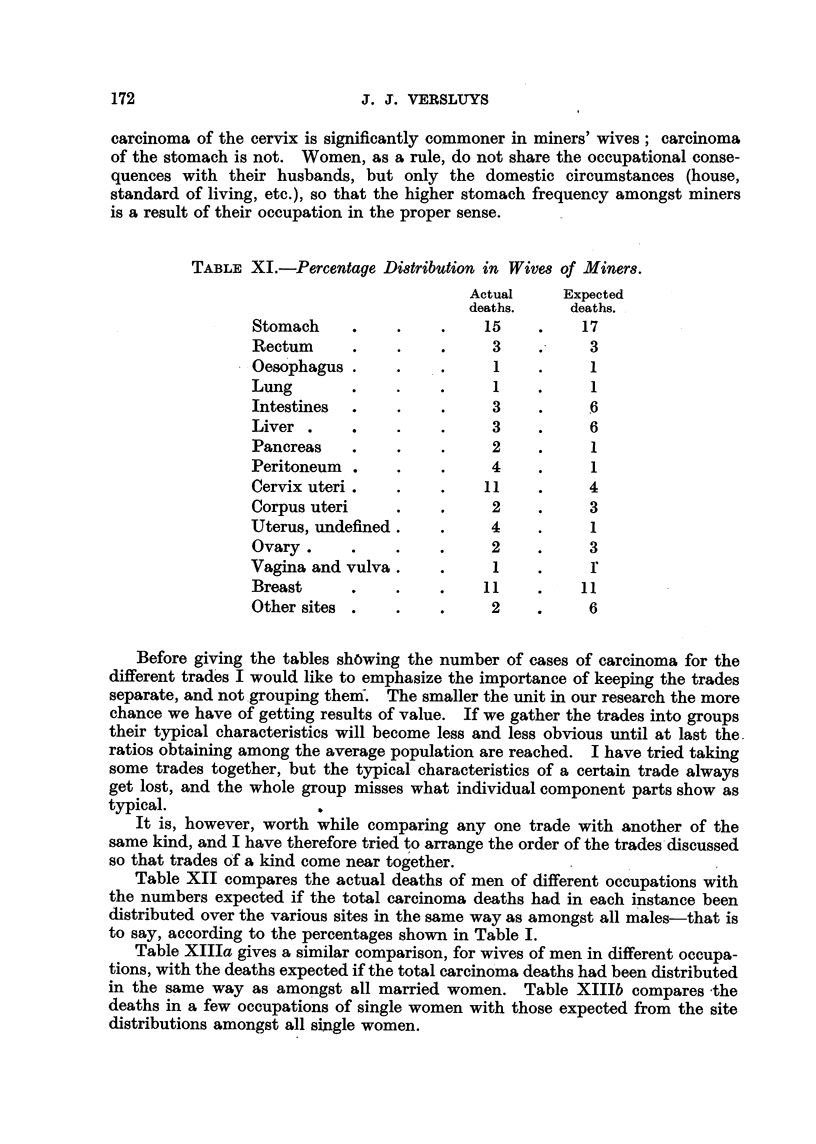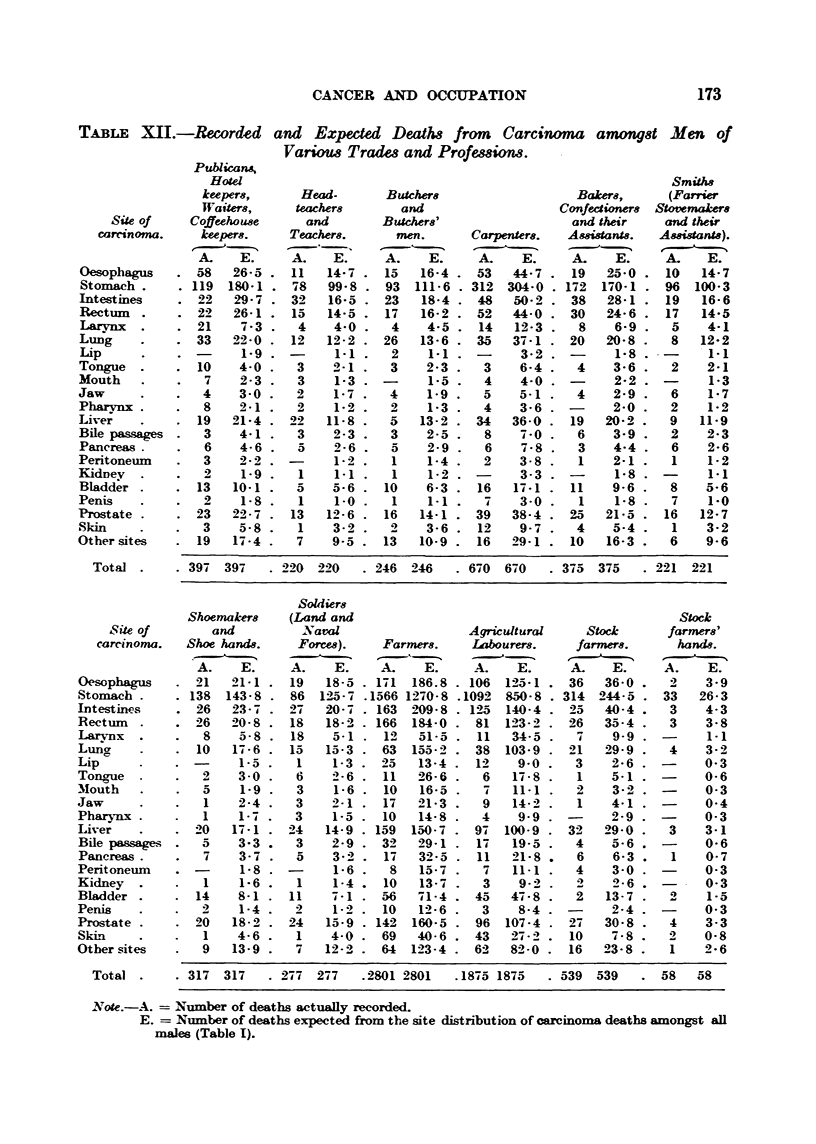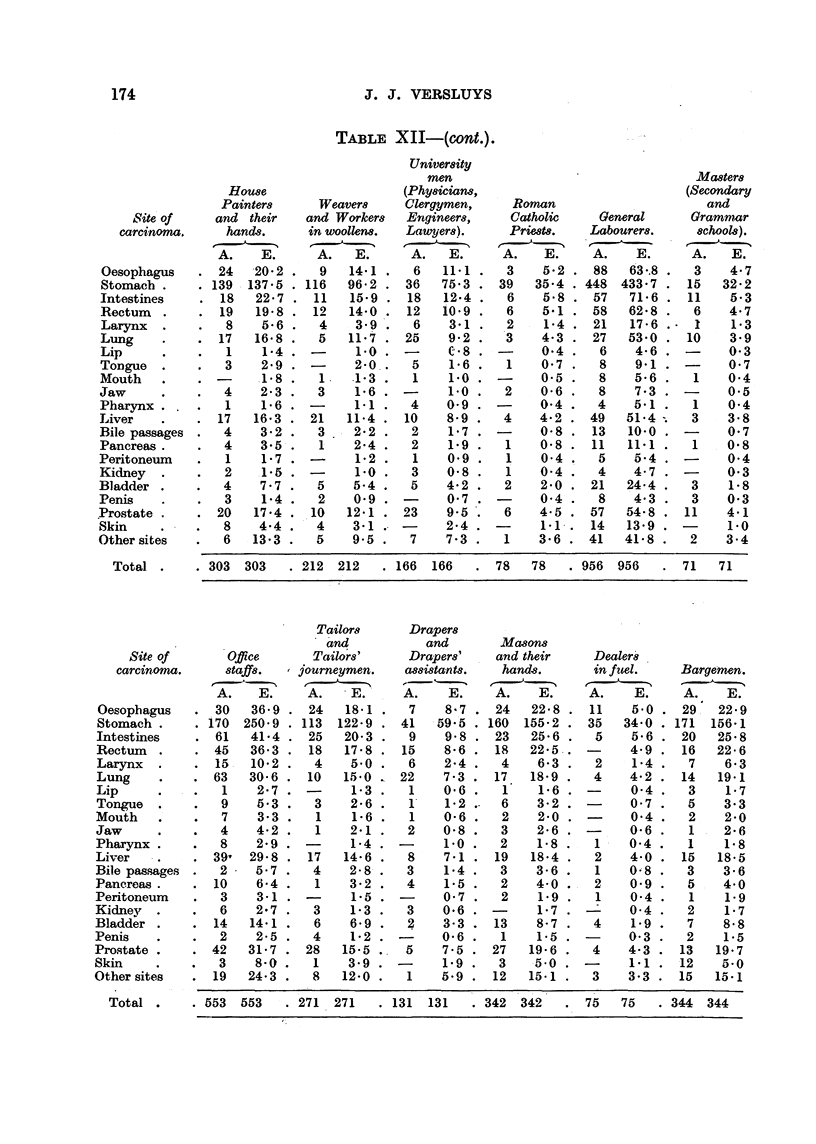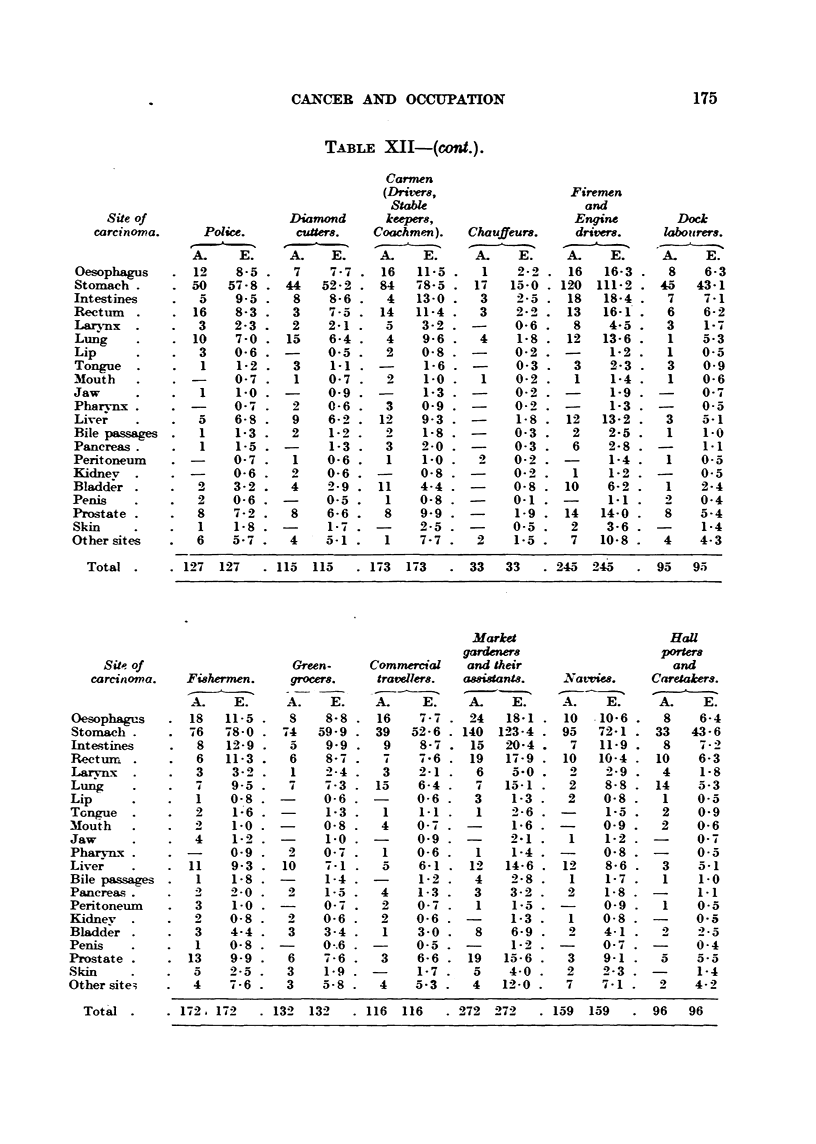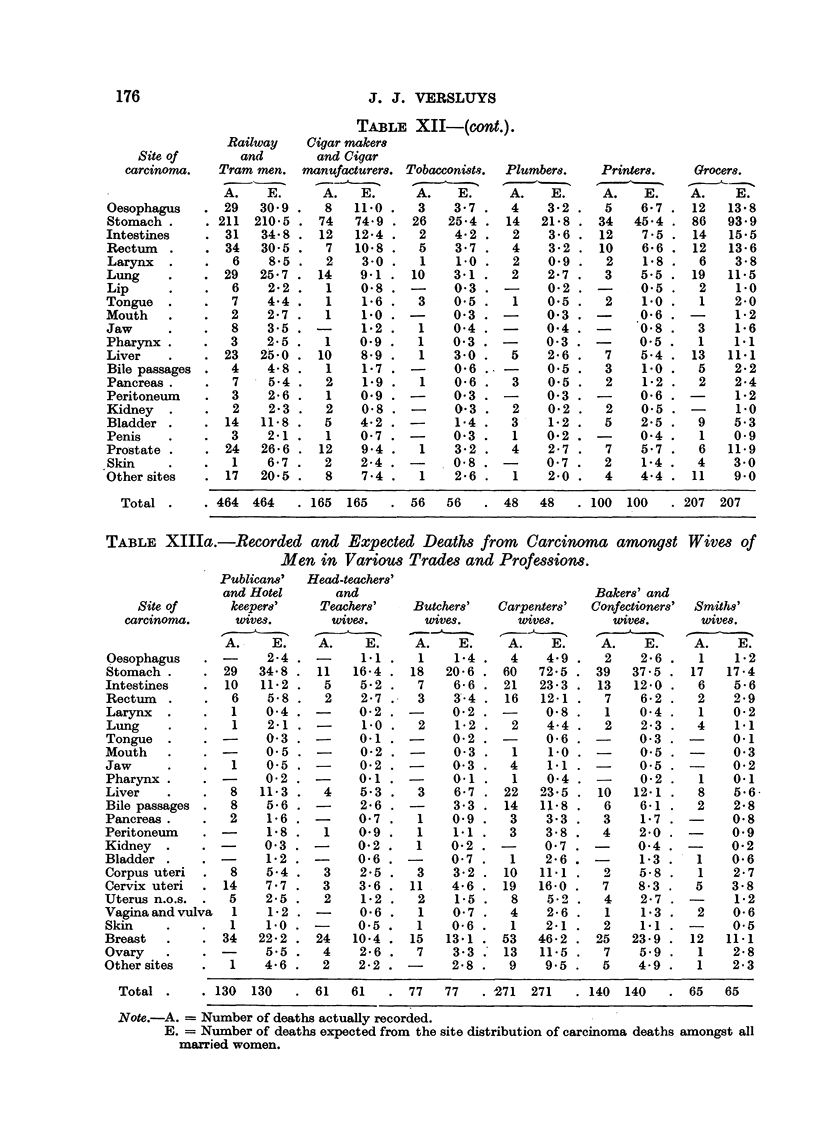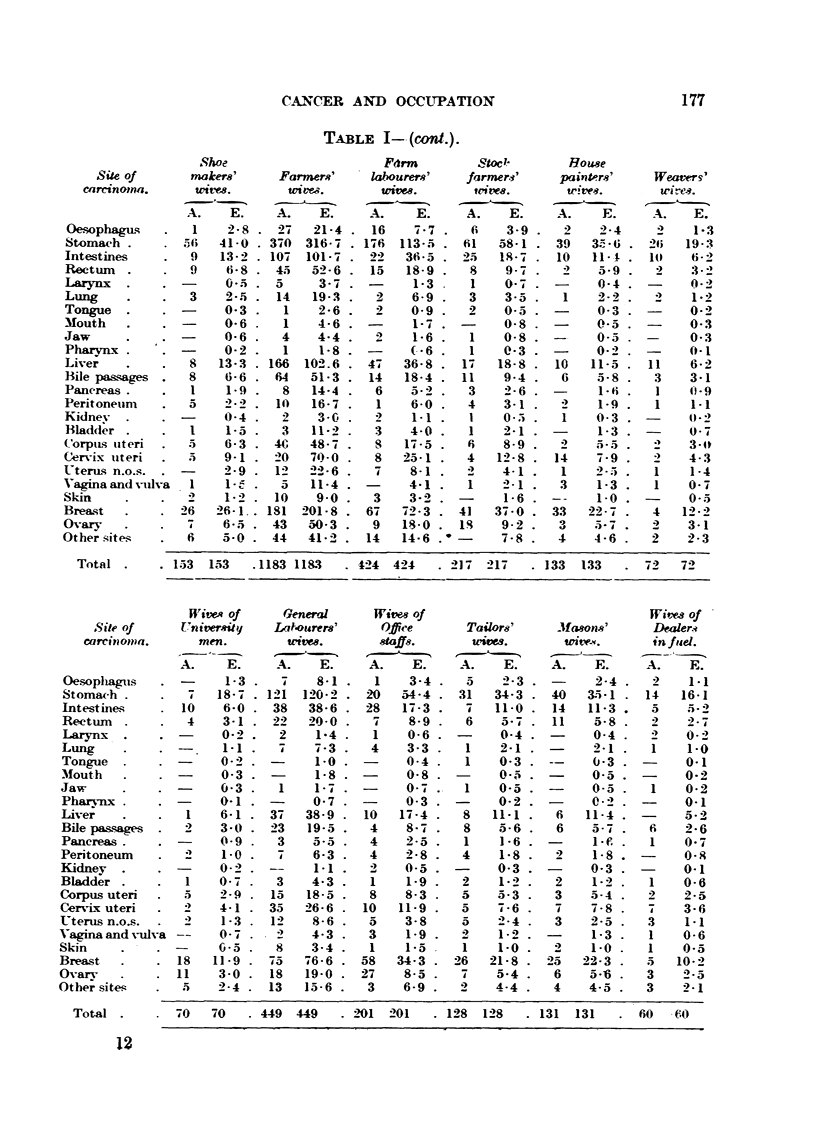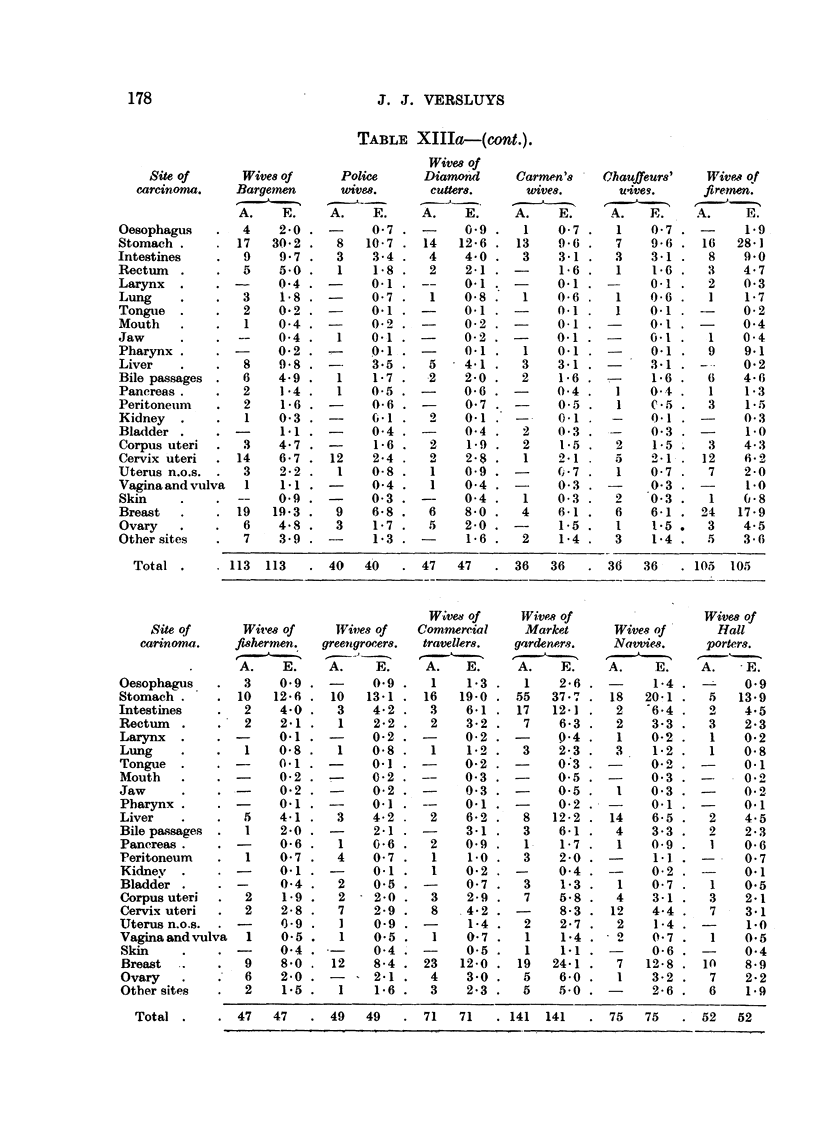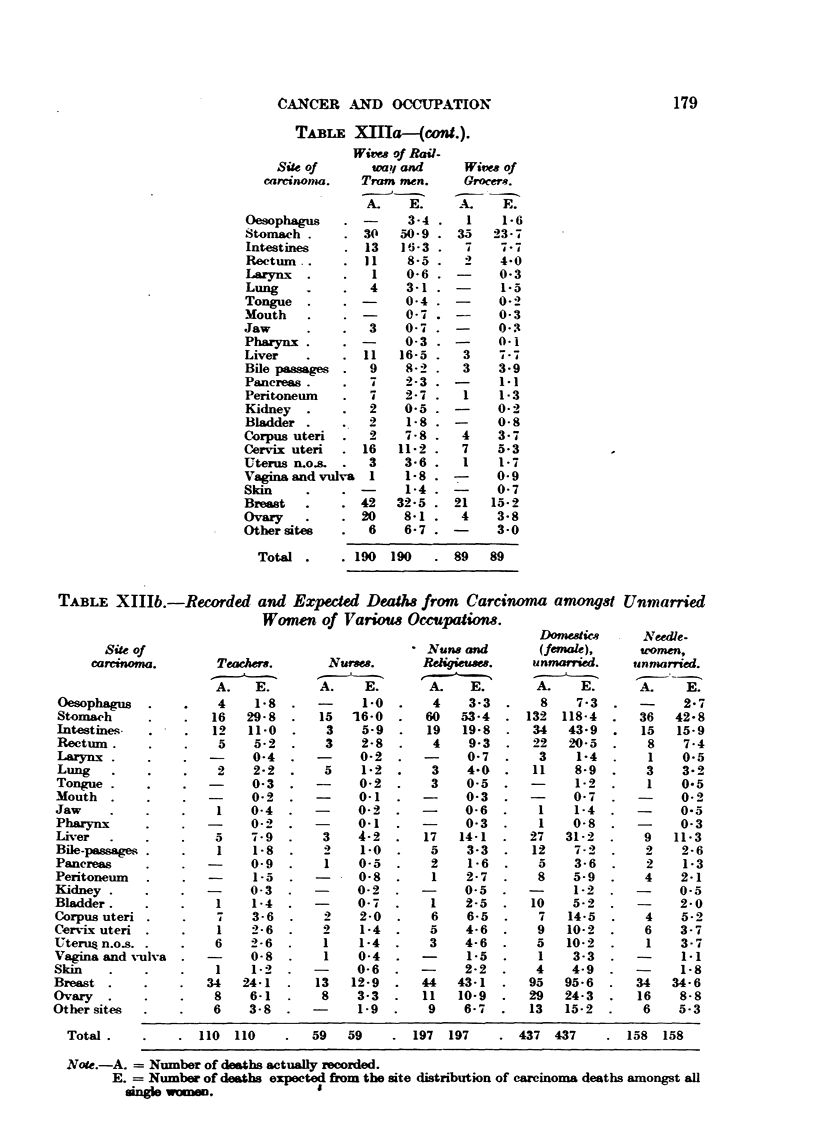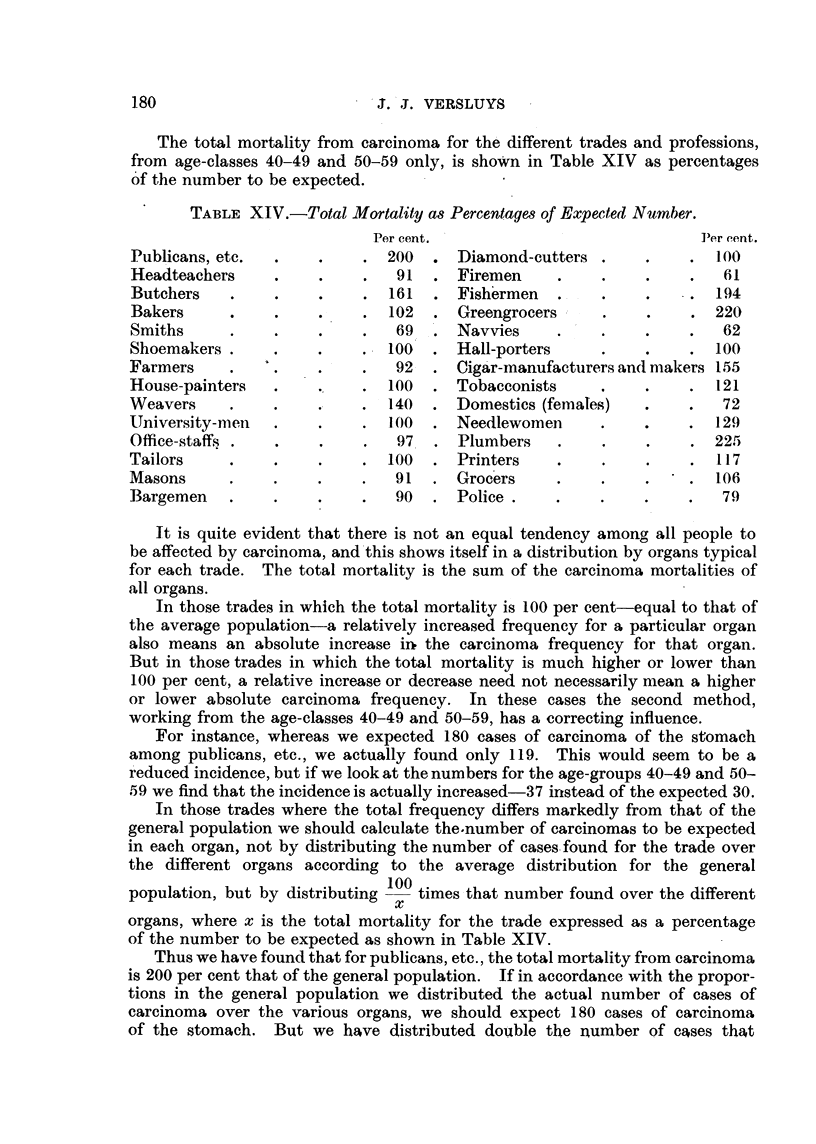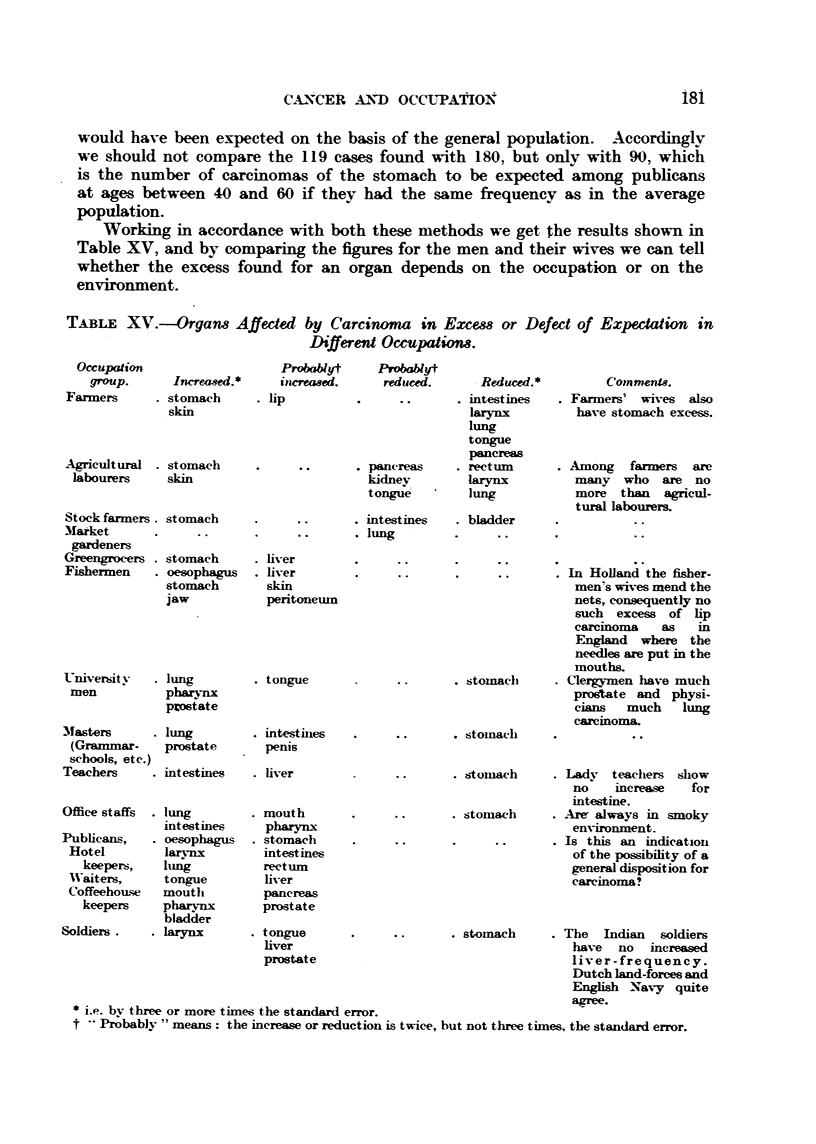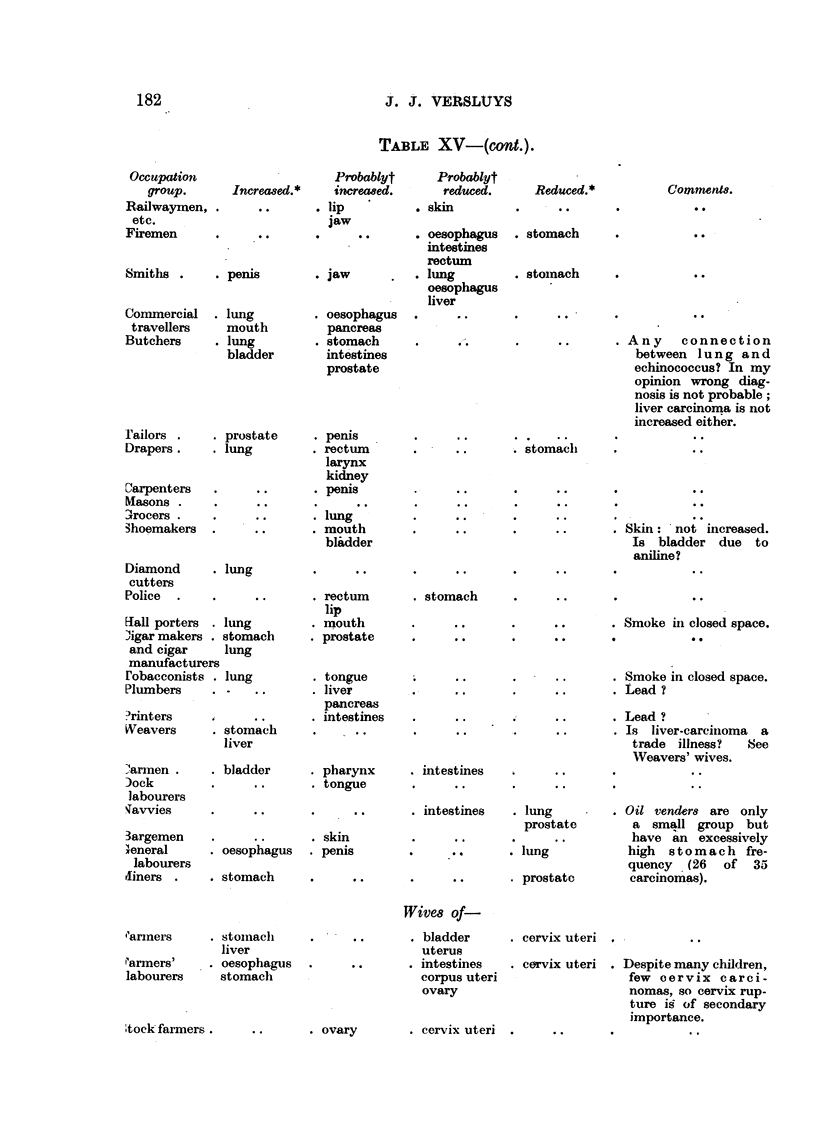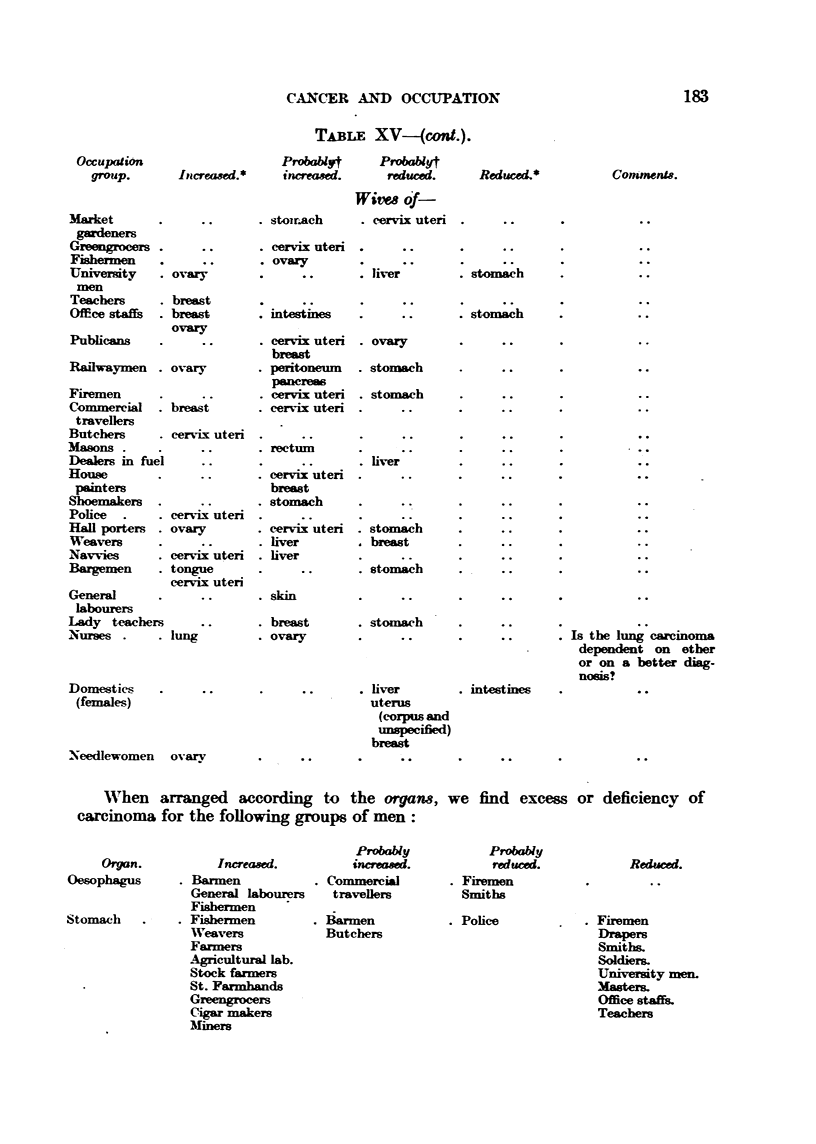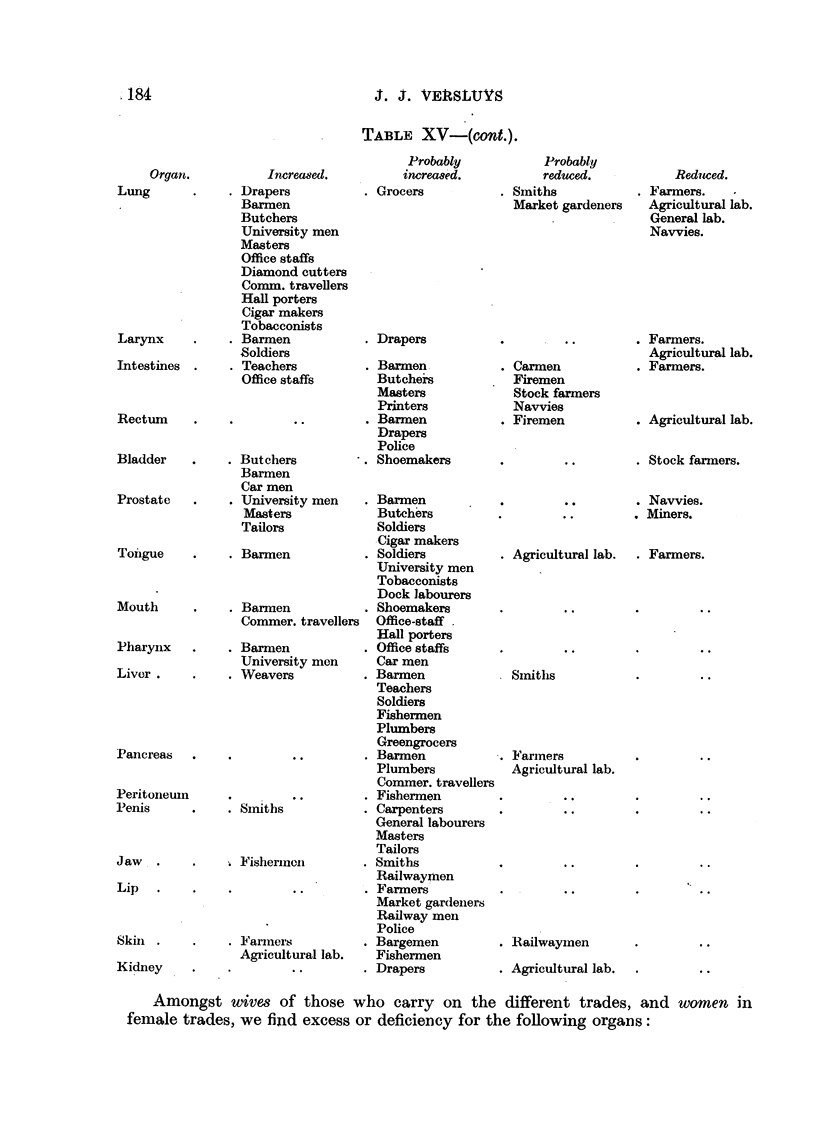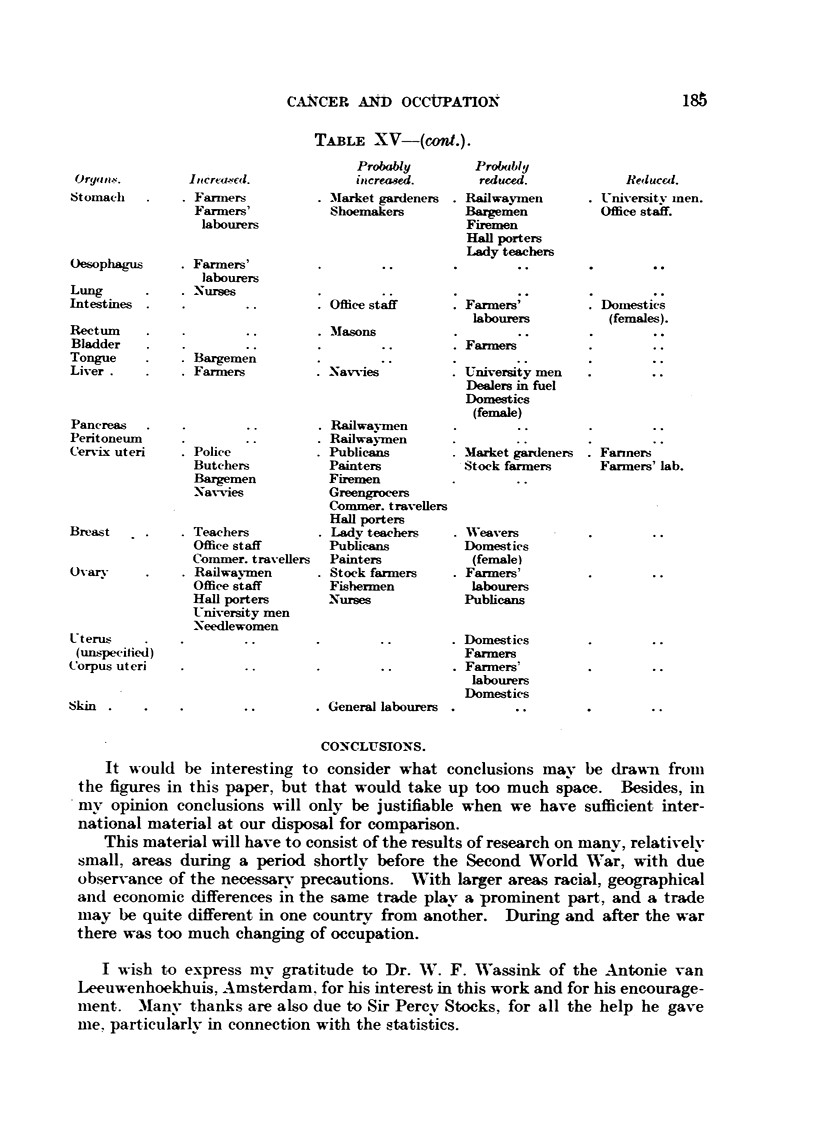# Cancer and Occupation in the Netherlands

**DOI:** 10.1038/bjc.1949.19

**Published:** 1949-06

**Authors:** J. J. Versluys


VOL. III | JUNE, 1949 | NO. 2

### CANCER AND OCCUPATION IN THE NETHERLANDS.

J. J. VERSLUYS.

*From Hillegom, Holland.*

Received for publication March 15, 1949.

THIS investigation into the mortality from carcinoma in the Netherlands during the years 1931–35 is based on the mortality-cards placed at our disposal for inspection by the courtesy of the Registrar-General.

In the Netherlands a card for each deceased person is filed with the Registrar-General, which mentions, among other things, the following particulars :

1. Male or female.
2. Married, single, divorced, widow(er).
3. Date of birth.
4. Date of death.
5. Occupation of the deceased.
6. Former occupation of the deceased.
7. Occupation of the head of the family (if the deceased himself had no occupation).
8. Cause of death (stated by the physician to the Registrar of births, deaths and marriages).
9. Cause of death (as stated by the physician under bond of secrecy to the Medical Offices attached to the Registrar-General).
10. Diseases from which the deceased suffered, but which were not the cause of death.

### TOTAL NUMBER OF DEATHS FROM CARCINOMA.

The total number of deaths from malignant tumours in the years 1931–35 amounted to 51,124. During this period there were 49,245 deaths from carcinoma, so that the total number of sarcomas, etc., was 1879, or less than 4 per cent of all malignant tumours.

More women than men die from carcinoma (25,927 women to 23,318 men), but on the other hand there are more women over the age of 40 than men. Taking into consideration the difference in number and the different age-grouping between men and women we find a somewhat different ratio. If there had been equal numbers of men and women, and equal numbers in the age-groups, on an average the male mortality would have amounted to 24,855. The number of female carcinoma deaths (only the deaths over 30 years of age are computed here) was 25,760. The difference is therefore much smaller (905 instead of 2609) than with the uncorrected figures.

TABLE I.—*Incidence of Carcinoma in the Various Organs of Men and Women.*

| | Men. | | Women. | |
|---|---|---|---|---|
| | Number. | Percentage of total. | Number. | Percentage of total. |
| Stomach | 10,579 | 45·37 | 7559 | 29·16 |
| Rectum | 1,533 | 6·57 | 1239 | 4·78 |
| Oesophagus | 1,555 | 6·67 | 565 | 2·18 |
| Intestines | 1,747 | 7·49 | 2497 | 9·63 |
| Peritoneum | 131 | 0·56 | 334 | 1·29 |
| Tongue | 222 | 0·95 | 55 | 0·21 |
| Lip | 111 | 0·48 | 10 | 0·04 |
| Mouth | 138 | 0·59 | 37 | 0·14 |
| Pharynx | 122 | 0·53 | 35 | 0·14 |
| Jaw | 178 | 0·76 | 86 | 0·33 |
| Liver (specified as primary) | 19 | 0·08 | 22 | 0·08 |
| ,, (not so specified) | 1,236 | 5·30 | 2272 | 8·76 |
| Bile passages | 243 | 1·04 | 984 | 3·80 |
| Larynx | 430 | 1·84 | 77 | 0·30 |
| Lung | 1,291 | 5·54 | 369 | 1·42 |
| Prostate | 1,336 | 5·73 | — | — |
| Penis | 104 | 0·45 | — | — |
| Bladder | 594 | 2·55 | 278 | 1·07 |
| Testis | 119 | 0·51 | — | — |
| Kidney | 114 | 0·49 | 68 | 0·26 |
| Skin | 337 | 1·45 | 309 | 1·19 |
| Pancreas | 271 | 1·16 | 259 | 1·00 |
| Breast | 37 | 0·16 | 4146 | 15·98 |
| Cervix uteri | — | — | 1272 | 4·91 |
| Corpus uteri | — | — | 737 | 2·84 |
| Uterus (undefined) | — | — | 504 | 1·94 |
| Ovary | — | — | 1026 | 3·96 |
| Vagina and vulva | — | — | 258 | 1·00 |
| Group 53 | 871 | 3·73 | 929 | 3·59 |
| All sites | 23,318 | 100·00 | 25,927 | 100·00 |

(Group 53 consists of carcinomas of sites which are only rarely affected).

The percentage distribution according to organ affected in men forms the basis for calculation of the expected deaths in Table XII.

Table II shows approximately the relative frequency of carcinoma of various sites with increasing age—approximately, for the percentage figures have this fault, that if one frequency is falling, the other percentages automatically rise; no value therefore should be attached to small differences. Only those organs which are affected with significant frequency are included in the table.

### *Ratio of Males to Females.*

The comparative frequencies for the most important organs are shown in Table III.

In general one might say that, taking no account of the genital organs, the "organs of entry and exit" are more commonly affected in men, and other organs in women. Environment and occupation may, therefore, be expected to exert a greater influence in men.

### *Average Age at Death from Carcinoma of Each Site.*

For each organ we can assess not only the frequency with which it is attacked, but also the average age at which this occurs. We are accustomed to speak of an average age in the case of a phenomenon which occurs more frequently with increasing age, reaches a maximum, and decreases again with further advancing years to become practically *nil*. Carcinoma is not a phenomenon of this kind;

TABLE II.—*Age Distribution of Carcinoma in Various Organs of Men and Women.*

| | Men. | | | |
|---|---|---|---|---|
| | 40–49. | 50–59. | 60–69. | 70 and over. |
| Stomach | 42·3 | 45·3 | 48·0 | 44·6 |
| Rectum | 7·4 | 7·0 | 5·9 | 6·6 |
| Oesophagus | 3·9 | 5·3 | 7·0 | 7·6 |
| Intestines | 7·4 | 7·4 | 6·9 | 7·8 |
| Liver | 4·9 | 5·3 | 5·2 | 5·4 |
| Larynx | 1·3 | 2·3 | 1·8 | 1·4 |
| Lung | 11·3 | 8·9 | 5·9 | 2·8 |
| Prostate | 1·2 | 2·2 | 5·3 | 8·4 |
| Bladder | 1·5 | 2·0 | 2·0 | 3·3 |
| Skin | 0·7 | 0·9 | 1·0 | 2·1 |
| Other sites | 18·1 | 13·4 | 11·0 | 10·0 |

| | Women. | | | |
|---|---|---|---|---|
| | 40–49. | 50–59. | 60–69. | 70 and over. |
| Stomach | 14·5 | 20·9 | 30·8 | 38·0 |
| Rectum | 4·2 | 4·6 | 4·6 | 5·0 |
| Oesophagus | 1·1 | 1·5 | 2·0 | 3·1 |
| Intestines | 5·4 | 6·8 | 9·9 | 12·3 |
| Gall-bladder | 2·3 | 3·9 | 4·8 | 3·6 |
| Liver | 4·6 | 7·0 | 9·9 | 10·5 |
| Lung | 1·9 | 1·8 | 1·6 | 0·7 |
| Breast | 31·4 | 22·9 | 13·7 | 8·3 |
| Ovary | 7·9 | 5·9 | 3·4 | 1·6 |
| Cervix uteri | 11·1 | 7·2 | 3·5 | 2·1 |
| Corpus uteri | 3·7 | 4·0 | 3·0 | 1·6 |
| Other sites | 11·9 | 13·5 | 15·8 | 13·2 |

TABLE III.—*Comparative Frequencies.*

| | Male. | Female. |
|---|---|---|
| Stomach | 1·40 | 1 |
| Rectum | 1·24 | 1 |
| Oesophagus | 2·75 | 1 |
| Intestines | 0·70 | 1 |
| Peritoneum | 0·39 | 1 |
| Tongue | 4·04 | 1 |
| Lip | 11·10 | 1 |
| Mouth | 3·73 | 1 |
| Pharynx | 3·49 | 1 |
| Liver | 0·53 | 1 |
| Bile passages | 0·25 | 1 |
| Larynx | 5·58 | 1 |
| Lung | 3·50 | 1 |
| Bladder | 2·14 | 1 |
| Skin | 1·09 | 1 |
| Pancreas | 1·05 | 1 |

a rise does indeed occur with increasing age, but the incidence continues to increase and the subsequent fall does not occur. Yet we can still speak of an average age, computed as follows :

Suppose we find in the age-class 30–39 *a* cases of carcinoma ; in the age-class 40–49, *b* cases ; in the class 50–59, *c* ; in the class 60–69, *d* ; and in the class 70 and over, *e* cases of carcinoma. Then the average age of these cases of carcinoma will be approximately :

$$\frac{(a \times 35) + (b \times 45) + (c \times 55) + (d \times 65) + (e \times 80)}{a + b + c + d + e}$$

The average ages at death for carcinomas in various organs of men and women computed in this way are shown in Table IV.

TABLE IV.—*Average Age at Death.*

| | Men. | Women. |
|---|---|---|
| Cervix uteri | — | 56·1 |
| Ovary | — | 57·2 |
| Breast | — | 58·6 |
| Lung | 61·8 | 60·3 |
| Corpus uteri | — | 60·7 |
| Pancreas | 62·7 | 61·4 |
| Rectum | 66·4 | 65·1 |
| Bile passages | 69·7 | 66·2 |
| Intestines | 67·6 | 67·9 |
| Stomach | 67·8 | 68·4 |
| Liver | 68·2 | 68·0 |
| Oesophagus | 70·0 | 69·7 |
| Bladder | 71·0 | 69·8 |
| Prostate | 71·8 | — |
| Skin | 72·1 | 72·6 |
| Penis | 72·4 | — |

From this it appears that :

(1) In women carcinomas of the reproductive organs have the youngest average age, but in men they have the highest. (The average age of tumours of the testis cannot be calculated, because on the mortality cards they are not clearly divided into carcinomas, mixed tumours, seminomas, etc.).

(2) The average for other organs in men and women is about the same, except for the bile passages ; and the order of the organs, arranged according to their average and excepting the bile passages, is the same.

*Death Rates by Age-classes for the Different Organs.*

For each site we have calculated the number of deaths per year per 100,000 living people belonging to each age-class. The results are shown in Table V.

From this table it appears that :

(1) The total death-rates in the three lowest age-groups are higher for women than for men ; but in the two highest age-groups they are higher for men than for women.

(2) Although in general the frequency of carcinoma increases as age advances, in some sites the frequency decreases with advancing years (ovary and lung in

TABLE V.—*Deaths per Year per 100,000 Living in each Age-class.*

| | Men. | | | | |
|---|---|---|---|---|---|
| | 30–39. | 40–49. | 50–59. | 60–69. | 70 and over. |
| All sites | 15·57 | 61·64 | 219·54 | 640·08 | 1363·19 |
| Stomach | 5·66 | 27·19 | 99·71 | 308·01 | 608·33 |
| Oesophagus | — | 2·47 | 11·61 | 45·14 | 104·16 |
| Rectum | 1·76 | 4·71 | 15·58 | 37·97 | 87·50 |
| Intestines | 1·59 | 4·71 | 16·14 | 44·30 | 106·93 |
| Liver | — | 3·14 | 11·89 | 33·75 | 74·60 |
| Lung | 1·23 | 7·19 | 19·59 | 37·97 | 38·88 |
| Larynx | — | — | 5·38 | 11·39 | 20·13 |
| Prostate | — | — | 5·09 | 34·17 | 114·58 |
| Bladder | — | 0·89 | 4·53 | 13·50 | 45·83 |
| Skin | — | — | 1·98 | 6·32 | 29·16 |

| | Women. | | | | |
|---|---|---|---|---|---|
| | 30–39. | 40–49. | 50–59. | 60–69. | 70 and over. |
| All sites | 28·74 | 113·60 | 294·52 | 610·40 | 1154·03 |
| Breast | 7·99 | 35·79 | 67·39 | 84·00 | 96·89 |
| Ovary | 1·87 | 9·11 | 17·53 | 21·25 | 18·63 |
| Cervix uteri | 3·91 | 12·79 | 21·09 | 21·60 | 24·22 |
| Corpus uteri | — | 4·34 | 12·05 | 18·80 | 19·31 |
| Stomach | 4·93 | 16·48 | 61·91 | 188·00 | 439·13 |
| Rectum | 1·70 | 4·77 | 13·69 | 28·40 | 38·38 |
| Oesophagus | — | 1·30 | 4·38 | 12·40 | 36·64 |
| Intestine | 1·87 | 6·29 | 20·23 | 60·40 | 142·85 |
| Liver | — | 5·42 | 20·82 | 60·40 | 122·35 |
| Gall-bladder | — | 3·81 | 11·50 | 29·60 | 42·23 |
| Lung | — | 2·17 | 5·20 | 10·00 | 8·69 |

women). In some sites (lung in men, corpus uteri, cervix and breast) the increase of frequency in the highest age-groups becomes small, while in others (prostate, intestine, oesophagus, bladder and skin) the increment of frequency is always rising.

If it were only the ovary, breast and corpus uteri which showed a diminishing rate of increase, one might think of a functional decline of those organs, but at 60 the lungs are surely not functionally worn out. It may be that the carcinoma frequency of every organ has its rise, culminating point and fall; but for some organs (prostate, intestines, etc.) people do not live long enough in sufficient numbers to demonstrate this decrease in frequency. A decrease in frequency of carcinoma at a higher age, as some investigators suggest, need not be ascribed to wrong diagnosis or less profound medical interest in the older individual.

TABLE VI.—*Percentage Unmarried.*

| | Men. | | | | |
|---|---|---|---|---|---|
| | 40–49. | 50–59. | 60–69. | 70 and over. | All ages. |
| Intestines | — | 9·7 | 9·5 | 8·3 | — |
| Liver | — | 10·0 | 11·1 | 6·5 | — |
| Bile passages | — | — | — | — | 9·9 |
| Pancreas | — | — | — | — | 8·5 |
| Skin | — | — | — | — | 17·5 |
| Penis | — | — | — | — | 7·6 |
| Prostate | — | — | 7·6 | 5·4 | — |
| Bladder | — | — | 8·6 | 6·9 | — |
| Lung | 10·1 | 9·5 | 5·9 | 7·7 | — |
| Oesophagus | — | 11·7 | 12·3 | 8·2 | — |
| Rectum | 11·4 | 9·4 | 7·9 | 8·9 | — |
| Stomach | 13·2 | 10·7 | 10·1 | 8·5 | — |
| Larynx | — | 12·8 | 12·1 | 5·6 | — |

| | Women. | | | | |
|---|---|---|---|---|---|
| | 40–49. | 50–59. | 60–69. | 70 and over. | All ages. |
| Intestines | — | 13·8 | 15·1 | 13·2 | — |
| Bile passages | — | 5·2 | 3·6 | 7·6 | — |
| Breast | 16·8 | 19·9 | 20·6 | 21·6 | — |
| Cervix | 3·0 | 5·9 | 10·7 | 9·2 | — |
| Corpus uteri | — | 13·2 | 16·1 | 21·0 | — |
| Ovary | 20·5 | 21·0 | 17·3 | 20·6 | — |
| Stomach | 16·7 | 14·9 | 11·8 | 12·8 | — |
| Lung | — | 21·6 | 23·8 | 12·5 | — |
| Rectum | — | 13·6 | 14·9 | 12·1 | — |
| Vagina and vulva | — | — | — | — | 9·7 |
| Liver | 17·0 | 13·6 | 9·9 | 9·5 | — |
| Bladder | — | — | — | — | 15·5 |
| Skin | — | — | — | — | 14·8 |
| Pancreas | — | — | — | — | 11·9 |
| Oesophagus | — | — | — | — | 10·7 |

*Influence of married state.*

The percentages of unmarried people in the average populations (according to the 1930 census) were :

| | 40–49. | 50–59. | 60–69. | 70 and over. |
|---|---|---|---|---|
| Men | 11·2 | 10·3 | 10·2 | 8·8 |
| Women | 15·6 | 14·2 | 13·8 | 12·5 |

Table VI shows for every age-class the percentage unmarried of the people who died from carcinoma of the various organs.

Exercising the necessary caution, we may infer from Table VI that—

(1) Carcinomas of the prostate and penis are found more frequently in married people (including those that have been married), and carcinoma of the skin in unmarried men.

(2) Carcinomas of the breast, body of uterus, ovary and lung occur especially among unmarried women, as also of the bladder and skin in a lesser degree, whereas cancer of the vagina, cervix and gall-bladder are distinctly commoner in married women.

Table VII.—*Incidence of Carcinoma in the Various Organs of Married and Single Women.*

| | Married. | | Single. | |
|---|---|---|---|---|
| | Number. | Percentage of total. | Number. | Percentage of total. |
| Oesophagus | 234 | 1·81 | 57 | 1·68 |
| Stomach | 3,466 | 26·77 | 917 | 27·09 |
| Intestines | 1,113 | 8·60 | 340 | 10·05 |
| Rectum | 576 | 4·45 | 158 | 4·70 |
| Larynx | 40 | 0·31 | 11 | 0·33 |
| Lung | 211 | 1·63 | 69 | 2·03 |
| Tongue | 28 | 0·22 | 9 | 0·27 |
| Mouth | 51 | 0·39 | 5 | 0·15 |
| Jaw | 48 | 0·37 | 11 | 0·33 |
| Pharynx | 20 | 0·15 | 6 | 0·18 |
| Liver | 1,123 | 8·67 | 242 | 7·15 |
| Bile passages | 563 | 4·34 | 56 | 1·65 |
| Pancreas | 158 | 1·22 | 28 | 0·83 |
| Peritoneum | 182 | 1·41 | 46 | 1·36 |
| Kidney | 32 | 0·25 | 9 | 0·27 |
| Bladder | 123 | 0·95 | 43 | 1·27 |
| Corpus uteri | 533 | 4·12 | 112 | 3·31 |
| Cervix uteri | 766 | 5·92 | 79 | 2·33 |
| Uterus n.o.s. | 247 | 1·91 | 79 | 2·33 |
| Vagina and vulva | 124 | 0·96 | 24 | 0·71 |
| Skin | 98 | 0·76 | 38 | 1·12 |
| Breast | 2,209 | 17·06 | 740 | 21·87 |
| Ovary | 550 | 4·25 | 188 | 5·56 |
| Other sites | 453 | 3·48 | 117 | 3·45 |
| All sites | 12,948 | 100·00 | 3384 | 100·00 |

(3) Carcinomas of the rectum, stomach and intestines show little association with wedlock.

We may note that the average age of mammary carcinoma is 58·6. Although after 45 there is practically no difference in the circumstances of the breast in married and unmarried women, yet in the higher age-groups the difference in frequency between the married and unmarried persists or is even greater. In my opinion this points to the existence of a long latent period, which we know exists in certain occupational cancers. The same holds good for carcinoma of the cervix, body of uterus and ovary.

The percentage distributions according to the organ affected in married and single women are shown in Table VII, and these form the bases for calculation of the expected deaths in Tables XIII*a* and XIII*b* respectively.

*Carcinoma in towns.*

Taking into account the age-group structure and the ratio between married and unmarried people in the towns, we can calculate the total mortality which might be expected if the mortality in towns were equal to that of the average population for the different sites. We should expect 12,875 deaths, whereas in reality we find 14,193 deaths, that is, an excess of 10·3 per cent. If we compute the mortality of the different organs in this way, it appears that in the big towns (which had more than 100,000 inhabitants on January 1, 1931) stomach and skin carcinoma mortalities are considerably lower, but for the tongue, larynx, lung, uterus, ovary and breast they are considerably higher.

The percentage distributions of the organs affected in the big towns and in the country, for men and women together, are shown in Table VIII.

TABLE VIII.—*Percentage Distributions in Big Towns and in Country.*

| | Town. | Country. |
|---|---|---|
| Mouth cavity | 1·8 | 1·5 |
| Tongue | 0·8 | 0·4 |
| Oesophagus | 4·2 | 4·1 |
| Stomach | 29·8 | 38·4 |
| Rectum | 5·8 | 5·1 |
| Liver, gall-bladder, etc. | 7·9 | 10·2 |
| Pancreas | 1·2 | 0·9 |
| Peritoneum | 1·1 | 0·8 |
| Intestines | 9·2 | 8·0 |
| Larynx | 1·3 | 0·8 |
| Lung and mediastinum | 4·7 | 2·9 |
| Uterus | 7·0 | 4·3 |
| Ovary | 2·5 | 1·9 |
| Breast | 9·7 | 7·7 |
| Male urogenital | 5·3 | 4·5 |
| Skin | 0·8 | 1·6 |
| Other sites | 6·9 | 6·9 |

*Jews and carcinoma.*

The distribution of deaths over the different organs in Jews is shown in Table IX.

We find a low figure for gastric carcinoma among Jews and high figures for carcinomas of the intestines, rectum, lung and bladder. Among Jewesses we find a low incidence for carcinomas of the stomach, bile passages and cervix, and a high incidence for carcinomas of the breast, ovary, intestines and kidneys.

It would be more correct to compare the Jews, not with the average population, but with that of the big towns, for 80·4 per cent and 80·7 per cent of Jews and Jewesses respectively live in the big towns. If we do so, we find an increased incidence for carcinomas of the liver, gall-bladder, intestines, rectum, lung, ovary and breast, and a decrease for the mouth, oesophagus, stomach, uterus and skin.

From the age-group distribution of Jews and Jewesses we should expect 228 and 296 cases of carcinoma respectively. We find, however, 246 and 368, that is to say 7·9 per cent and 24·3 per cent more than in the average population. So we must conclude that whilst Jews yield about as many cases of carcinoma as their environment leads us to expect, Jewesses on the other hand yield considerably more.

TABLE IX.—*Distribution of Deaths over Different Organs in Jews.*

| | Men. | | Women. | |
|---|---|---|---|---|
| | Actual. | Expected. | Actual. | Expected. |
| Oesophagus | 15 | 16·4 | 6 | 8·0 |
| Stomach | 64 | 111·6 | 70 | 107·3 |
| Intestines | 33 | 18·4 | 53 | 35·4 |
| Rectum | 26 | 16·2 | 18 | 17·6 |
| Larynx | 4 | 4·5 | 1 | 1·1 |
| Lung | 30 | 13·6 | 7 | 5·2 |
| Lip | 1 | 1·2 | — | 0·1 |
| Mouth | 2 | 1·5 | — | 0·5 |
| Tongue | 4 | 2·3 | — | 0·8 |
| Jaw | 3 | 1·9 | 1 | 1·2 |
| Pharynx | 1 | 1·3 | — | 0·5 |
| Liver | 13 | 13·2 | 29 | 33·3 |
| Bile-passages | 5 | 2·6 | 7 | 14·0 |
| Pancreas | 4 | 2·9 | 2 | 3·7 |
| Peritoneum | 1 | 1·4 | 4 | 4·7 |
| Kidney | 3 | 1·2 | 5 | 0·3 |
| Bladder | 12 | 6·3 | 7 | 3·9 |
| Prostate | 15 | 14·1 | — | — |
| Penis | — | 1·1 | — | — |
| Corpus uteri | — | — | 17 | 10·5 |
| Cervix uteri | — | — | 9 | 18·1 |
| Uterus (undefined) | — | — | 6 | 7·1 |
| Vagina and vulva | — | — | 4 | 3·7 |
| Breast | — | — | 81 | 58·8 |
| Ovary | — | — | 28 | 14·6 |
| Skin | 1 | 3·6 | 1 | 4·4 |
| Other sites | 9 | 10·9 | 12 | 13·2 |
| | 246 | 246 | 368 | 368 |

*Carcinoma and trade or profession.*

We now come to the real object of this research—to trace the influence of trade or profession on the origin and the localization of carcinoma. Our research covers a wider field than that of the so-called occupational cancers, and all that has gone before is necessary to enable us to interpret what follows.

Before we can proceed, we shall have to examine some problems a little more closely. This will be facilitated by taking a specific occupation as an example, and I have chosen coal-mining.

The mortality cards show us—

A. How many miners died of carcinoma from 1931 to 1935, their ages, and the sites of their carcinomas.

B. How many people who had formerly been miners died of carcinoma from 1931 to 1935, their ages, and the sites of their carcinomas.

The census of 1930 (there was no census in 1940 on account of the German occupation, consequently an interpolation is impossible for the years between 1930 and 1940) shows us—

How many miners there were in the Netherlands and their ages, but nothing about ex-miners.

Now if we take groups A and B together we include all miners who got carcinoma while still working or after leaving the trade, with their ages, but their distribution over the various age-classes cannot be compared with the census of 1930, which gives no information about ex-miners. We can best get over this difficulty by expressing the actual deaths for each site as percentages of those for all sites, and comparing the distribution for each occupation with that for all males ; the drawbacks to this plan are that the percentages are mutually dependent, and if the percentage of one side rises, the other percentages automatically fall, and also that we cannot form an opinion about the absolute frequency of carcinoma in the different trades.

On the other hand, if we only take group A there are the difficulties that the number of cases of carcinoma for each trade will be smaller, and that miners, especially the older ones, leave off working when they fall ill with carcinoma, and consequently on the mortality cards they are stated as "occupation—none." The latter difficulty might be overcome by considering only those miners who died under the age of 60 (before this age they cannot give up their trade for financial reasons) ; but then the numbers available become much smaller, and those carcinomas whose average age lies over 60 will be left out of account.

In my opinion the best way is to use both methods, the latter with the correction that only the cases in the age-groups 30–39, 40–49 and 50–59 are taken into account. In this way we get a comparison of percentages of the greatest possible number of cases, and absolute figures besides.

The percentage distribution of carcinomas over the various organs in coal-miners, both in employment and formerly employed, is shown in Table X.

The column "to be expected" in Table X shows how the distribution of the cases of carcinoma in a certain grade over the different organs would be, if the distribution we found for the whole male (or female) population applied.

TABLE X.—*Percentage Distribution of Carcinomas over the Organs in Coal-miners.*

| | In employment. | Formerly employed. | Total. | To be expected. |
|---|---|---|---|---|
| Stomach | 39 | 29 | 68 | 45·4 |
| Rectum | 2 | 1 | 3 | 6·6 |
| Oesophagus | 3 | 1 | 4 | 6·7 |
| Lung | 5 | 3 | 8 | 5·5 |
| Intestines | 1 | 2 | 3 | 7·5 |
| Liver | 2 | 4 | 6 | 5·4 |
| Bile-passages | — | 2 | 2 | 1·0 |
| Larynx | — | 1 | 1 | 1·8 |
| Peritoneum | — | 1 | 1 | 0·6 |
| Prostate | — | 1 | 1 | 5·7 |
| Bladder | — | 1 | 1 | 2·5 |
| Other sites | 1 | 1 | 2 | 11·3 |
| | 53 | 47 | 100 | 100 |

To decide whether the differences found are real or not the average error must be calculated. This has been done following the method of Dr. Bruno Scholz (*Methodik der medizinischen Erforschung*, page 133). Suppose we find a total of $x$ cases of carcinoma in a certain trade. Of these, $y$ cases are carcinomas of the stomach. Then the average error is $\sqrt{\frac{y \times (x - y)}{x}}$. When $y$ is small this is practically equal to $\sqrt{y}$.

This average error has always been taken into account in this research, and a difference is called real only when the percentages found and those to be expected differ by at least 3 times the average error.

We find for miners a high frequency of carcinoma of the stomach and a low prostate frequency. In the age-classes 30–39, 40–49 and 50–59 we find a total mortality of 50 cases, whereas we should expect 32, so that the total carcinoma mortality of coal miners is very high. The frequency of carcinoma of the stomach is greatly increased, for we find 33 cases instead of the 12 we should expect. The number of cases of carcinoma of the prostate in these age-classes is too small to reach a conclusion.

The question now arises, is this high stomach frequency amongst miners bound up with their trade or with their environment ; is it an " occupational cancer " or a " social cancer "? (Cramer). To answer this question we must study " wives of miners " (from the mortality cards of those married women where the trade of the head of the family is given as " coal miner." The married woman practically always lodges with her husband).

The percentage distribution of carcinoma over the various organs in " wives of miners " compared with the distribution in married women amongst the general population is shown in Table XI.

Taking the " average error " into account, we see from Table XI that only carcinoma of the cervix is significantly commoner in miners' wives; carcinoma of the stomach is not. Women, as a rule, do not share the occupational consequences with their husbands, but only the domestic circumstances (house, standard of living, etc.), so that the higher stomach frequency amongst miners is a result of their occupation in the proper sense.

TABLE XI.—*Percentage Distribution in Wives of Miners.*

| | Actual deaths. | Expected deaths. |
|---|---|---|
| Stomach | 15 | 17 |
| Rectum | 3 | 3 |
| Oesophagus | 1 | 1 |
| Lung | 1 | 1 |
| Intestines | 3 | 6 |
| Liver | 3 | 6 |
| Pancreas | 2 | 1 |
| Peritoneum | 4 | 1 |
| Cervix uteri | 11 | 4 |
| Corpus uteri | 2 | 3 |
| Uterus, undefined | 4 | 1 |
| Ovary | 2 | 3 |
| Vagina and vulva | 1 | 1 |
| Breast | 11 | 11 |
| Other sites | 2 | 6 |

Before giving the tables showing the number of cases of carcinoma for the different trades I would like to emphasize the importance of keeping the trades separate, and not grouping them. The smaller the unit in our research the more chance we have of getting results of value. If we gather the trades into groups their typical characteristics will become less and less obvious until at last the ratios obtaining among the average population are reached. I have tried taking some trades together, but the typical characteristics of a certain trade always get lost, and the whole group misses what individual component parts show as typical.

It is, however, worth while comparing any one trade with another of the same kind, and I have therefore tried to arrange the order of the trades discussed so that trades of a kind come near together.

Table XII compares the actual deaths of men of different occupations with the numbers expected if the total carcinoma deaths had in each instance been distributed over the various sites in the same way as amongst all males—that is to say, according to the percentages shown in Table I.

Table XIII*a* gives a similar comparison, for wives of men in different occupations, with the deaths expected if the total carcinoma deaths had been distributed in the same way as amongst all married women. Table XIII*b* compares the deaths in a few occupations of single women with those expected from the site distributions amongst all single women.

TABLE XII.—*Recorded and Expected Deaths from Carcinoma amongst Men of Various Trades and Professions.*

| *Site of carcinoma.* | *Publicans, Hotel keepers, Waiters, Coffeehouse keepers.* | | *Head-teachers and Teachers.* | | *Butchers and Butchers' men.* | | *Carpenters.* | | *Bakers, Confectioners and their Assistants.* | | *Smiths (Farrier Stovemakers and their Assistants).* | |
|---|---|---|---|---|---|---|---|---|---|---|---|---|
| | A. | E. | A. | E. | A. | E. | A. | E. | A. | E. | A. | E. |
| Oesophagus | 58 | 26·5 | 11 | 14·7 | 15 | 16·4 | 53 | 44·7 | 19 | 25·0 | 10 | 14·7 |
| Stomach | 119 | 180·1 | 78 | 99·8 | 93 | 111·6 | 312 | 304·0 | 172 | 170·1 | 96 | 100·3 |
| Intestines | 22 | 29·7 | 32 | 16·5 | 23 | 18·4 | 48 | 50·2 | 38 | 28·1 | 19 | 16·6 |
| Rectum | 22 | 26·1 | 15 | 14·5 | 17 | 16·2 | 52 | 44·0 | 30 | 24·6 | 17 | 14·5 |
| Larynx | 21 | 7·3 | 4 | 4·0 | 4 | 4·5 | 14 | 12·3 | 8 | 6·9 | 5 | 4·1 |
| Lung | 33 | 22·0 | 12 | 12·2 | 26 | 13·6 | 35 | 37·1 | 20 | 20·8 | 8 | 12·2 |
| Lip | — | 1·9 | — | 1·1 | 2 | 1·1 | — | 3·2 | — | 1·8 | — | 1·1 |
| Tongue | 10 | 4·0 | 3 | 2·1 | 3 | 2·3 | 3 | 6·4 | 4 | 3·6 | 2 | 2·1 |
| Mouth | 7 | 2·3 | 3 | 1·3 | — | 1·5 | 4 | 4·0 | — | 2·2 | — | 1·3 |
| Jaw | 4 | 3·0 | 2 | 1·7 | 4 | 1·9 | 5 | 5·1 | 4 | 2·9 | 6 | 1·7 |
| Pharynx | 8 | 2·1 | 2 | 1·2 | 2 | 1·3 | 4 | 3·6 | — | 2·0 | 2 | 1·2 |
| Liver | 19 | 21·4 | 22 | 11·8 | 5 | 13·2 | 34 | 36·0 | 19 | 20·2 | 9 | 11·9 |
| Bile passages | 3 | 4·1 | 3 | 2·3 | 3 | 2·5 | 8 | 7·0 | 6 | 3·9 | 2 | 2·3 |
| Pancreas | 6 | 4·6 | 5 | 2·6 | 5 | 2·9 | 6 | 7·8 | 3 | 4·4 | 6 | 2·6 |
| Peritoneum | 3 | 2·2 | — | 1·2 | 1 | 1·4 | 2 | 3·8 | 1 | 2·1 | 1 | 1·2 |
| Kidney | 2 | 1·9 | 1 | 1·1 | 1 | 1·2 | — | 3·3 | — | 1·8 | — | 1·1 |
| Bladder | 13 | 10·1 | 5 | 5·6 | 10 | 6·3 | 16 | 17·1 | 11 | 9·6 | 8 | 5·6 |
| Penis | 2 | 1·8 | 1 | 1·0 | 1 | 1·1 | 7 | 3·0 | 1 | 1·8 | 7 | 1·0 |
| Prostate | 23 | 22·7 | 13 | 12·6 | 16 | 14·1 | 39 | 38·4 | 25 | 21·5 | 16 | 12·7 |
| Skin | 3 | 5·8 | 1 | 3·2 | 2 | 3·6 | 12 | 9·7 | 4 | 5·4 | 1 | 3·2 |
| Other sites | 19 | 17·4 | 7 | 9·5 | 13 | 10·9 | 16 | 29·1 | 10 | 16·3 | 6 | 9·6 |
| Total | 397 | 397 | 220 | 220 | 246 | 246 | 670 | 670 | 375 | 375 | 221 | 221 |

| *Site of carcinoma.* | *Shoemakers and Shoe hands.* | | *Soldiers (Land and Naval Forces).* | | *Farmers.* | | *Agricultural Labourers.* | | *Stock farmers.* | | *Stock farmers' hands.* | |
|---|---|---|---|---|---|---|---|---|---|---|---|---|
| | A. | E. | A. | E. | A. | E. | A. | E. | A. | E. | A. | E. |
| Oesophagus | 21 | 21·1 | 19 | 18·5 | 171 | 186.8 | 106 | 125·1 | 36 | 36·0 | 2 | 3·9 |
| Stomach | 138 | 143·8 | 86 | 125·7 | 1566 | 1270·8 | 1092 | 850·8 | 314 | 244·5 | 33 | 26·3 |
| Intestines | 26 | 23·7 | 27 | 20·7 | 163 | 209·8 | 125 | 140·4 | 25 | 40·4 | 3 | 4·3 |
| Rectum | 26 | 20·8 | 18 | 18·2 | 166 | 184·0 | 81 | 123·2 | 26 | 35·4 | 3 | 3·8 |
| Larynx | 8 | 5·8 | 18 | 5·1 | 12 | 51·5 | 11 | 34·5 | 7 | 9·9 | — | 1·1 |
| Lung | 10 | 17·6 | 15 | 15·3 | 63 | 155·2 | 38 | 103·9 | 21 | 29·9 | 4 | 3·2 |
| Lip | — | 1·5 | 1 | 1·3 | 25 | 13·4 | 12 | 9·0 | 3 | 2·6 | — | 0·3 |
| Tongue | 2 | 3·0 | 6 | 2·6 | 11 | 26·6 | 6 | 17·8 | 1 | 5·1 | — | 0·6 |
| Mouth | 5 | 1·9 | 3 | 1·6 | 10 | 16·5 | 7 | 11·1 | 2 | 3·2 | — | 0·3 |
| Jaw | 1 | 2·4 | 3 | 2·1 | 17 | 21·3 | 9 | 14·2 | 1 | 4·1 | — | 0·4 |
| Pharynx | 1 | 1·7 | 3 | 1·5 | 10 | 14·8 | 4 | 9·9 | — | 2·9 | — | 0·3 |
| Liver | 20 | 17·1 | 24 | 14·9 | 159 | 150·7 | 97 | 100·9 | 32 | 29·0 | 3 | 3·1 |
| Bile passages | 5 | 3·3 | 3 | 2·9 | 32 | 29·1 | 17 | 19·5 | 4 | 5·6 | — | 0·6 |
| Pancreas | 7 | 3·7 | 5 | 3·2 | 17 | 32·5 | 11 | 21·8 | 6 | 6·3 | 1 | 0·7 |
| Peritoneum | — | 1·8 | — | 1·6 | 8 | 15·7 | 7 | 11·1 | 4 | 3·0 | — | 0·3 |
| Kidney | 1 | 1·6 | 1 | 1·4 | 10 | 13·7 | 3 | 9·2 | 2 | 2·6 | — | 0·3 |
| Bladder | 14 | 8·1 | 11 | 7·1 | 56 | 71·4 | 45 | 47·8 | 2 | 13·7 | 2 | 1·5 |
| Penis | 2 | 1·4 | 2 | 1·2 | 10 | 12·6 | 3 | 8·4 | — | 2·4 | — | 0·3 |
| Prostate | 20 | 18·2 | 24 | 15·9 | 142 | 160·5 | 96 | 107·4 | 27 | 30·8 | 4 | 3·3 |
| Skin | 1 | 4·6 | 1 | 4·0 | 69 | 40·6 | 43 | 27·2 | 10 | 7·8 | 2 | 0·8 |
| Other sites | 9 | 13·9 | 7 | 12·2 | 64 | 123·4 | 62 | 82·0 | 16 | 23·8 | 1 | 2·6 |
| Total | 317 | 317 | 277 | 277 | 2801 | 2801 | 1875 | 1875 | 539 | 539 | 58 | 58 |

*Note.*—A. = Number of deaths actually recorded.
E. = Number of deaths expected from the site distribution of carcinoma deaths amongst all males (Table I).

TABLE XII—(*cont.*).

| *Site of carcinoma.* | *House Painters and their hands.* A. | E. | *Weavers and Workers in woollens.* A. | E. | *University men (Physicians, Clergymen, Engineers, Lawyers).* A. | E. | *Roman Catholic Priests.* A. | E. | *General Labourers.* A. | E. | *Masters (Secondary and Grammar schools).* A. | E. |
|---|---|---|---|---|---|---|---|---|---|---|---|---|
| Oesophagus | 24 | 20·2 | 9 | 14·1 | 6 | 11·1 | 3 | 5·2 | 88 | 63·8 | 3 | 4·7 |
| Stomach | 139 | 137·5 | 116 | 96·2 | 36 | 75·3 | 39 | 35·4 | 448 | 433·7 | 15 | 32·2 |
| Intestines | 18 | 22·7 | 11 | 15·9 | 18 | 12·4 | 6 | 5·8 | 57 | 71·6 | 11 | 5·3 |
| Rectum | 19 | 19·8 | 12 | 14·0 | 12 | 10·9 | 6 | 5·1 | 58 | 62·8 | 6 | 4·7 |
| Larynx | 8 | 5·6 | 4 | 3·9 | 6 | 3·1 | 2 | 1·4 | 21 | 17·6 | 1 | 1·3 |
| Lung | 17 | 16·8 | 5 | 11·7 | 25 | 9·2 | 3 | 4·3 | 27 | 53·0 | 10 | 3·9 |
| Lip | 1 | 1·4 | — | 1·0 | — | 0·8 | — | 0·4 | 6 | 4·6 | — | 0·3 |
| Tongue | 3 | 2·9 | — | 2·0 | 5 | 1·6 | 1 | 0·7 | 8 | 9·1 | — | 0·7 |
| Mouth | — | 1·8 | 1 | 1·3 | 1 | 1·0 | — | 0·5 | 8 | 5·6 | 1 | 0·4 |
| Jaw | 4 | 2·3 | 3 | 1·6 | — | 1·0 | 2 | 0·6 | 8 | 7·3 | — | 0·5 |
| Pharynx | 1 | 1·6 | — | 1·1 | 4 | 0·9 | — | 0·4 | 4 | 5·1 | 1 | 0·4 |
| Liver | 17 | 16·3 | 21 | 11·4 | 10 | 8·9 | 4 | 4·2 | 49 | 51·4 | 3 | 3·8 |
| Bile passages | 4 | 3·2 | 3 | 2·2 | 2 | 1·7 | — | 0·8 | 13 | 10·0 | — | 0·7 |
| Pancreas | 4 | 3·5 | 1 | 2·4 | 2 | 1·9 | 1 | 0·8 | 11 | 11·1 | 1 | 0·8 |
| Peritoneum | 1 | 1·7 | — | 1·2 | 1 | 0·9 | 1 | 0·4 | 5 | 5·4 | — | 0·4 |
| Kidney | 2 | 1·5 | — | 1·0 | 3 | 0·8 | 1 | 0·4 | 4 | 4·7 | — | 0·3 |
| Bladder | 4 | 7·7 | 5 | 5·4 | 5 | 4·2 | 2 | 2·0 | 21 | 24·4 | 3 | 1·8 |
| Penis | 3 | 1·4 | 2 | 0·9 | — | 0·7 | — | 0·4 | 8 | 4·3 | 3 | 0·3 |
| Prostate | 20 | 17·4 | 10 | 12·1 | 23 | 9·5 | 6 | 4·5 | 57 | 54·8 | 11 | 4·1 |
| Skin | 8 | 4·4 | 4 | 3·1 | — | 2·4 | — | 1·1 | 14 | 13·9 | — | 1·0 |
| Other sites | 6 | 13·3 | 5 | 9·5 | 7 | 7·3 | 1 | 3·6 | 41 | 41·8 | 2 | 3·4 |
| Total | 303 | 303 | 212 | 212 | 166 | 166 | 78 | 78 | 956 | 956 | 71 | 71 |

| *Site of carcinoma.* | *Office staffs.* A. | E. | *Tailors and Tailors' journeymen.* A. | E. | *Drapers and Drapers' assistants.* A. | E. | *Masons and their hands.* A. | E. | *Dealers in fuel.* A. | E. | *Bargemen.* A. | E. |
|---|---|---|---|---|---|---|---|---|---|---|---|---|
| Oesophagus | 30 | 36·9 | 24 | 18·1 | 7 | 8·7 | 24 | 22·8 | 11 | 5·0 | 29 | 22·9 |
| Stomach | 170 | 250·9 | 113 | 122·9 | 41 | 59·5 | 160 | 155·2 | 35 | 34·0 | 171 | 156·1 |
| Intestines | 61 | 41·4 | 25 | 20·3 | 9 | 9·8 | 23 | 25·6 | 5 | 5·6 | 20 | 25·8 |
| Rectum | 45 | 36·3 | 18 | 17·8 | 15 | 8·6 | 18 | 22·5 | — | 4·9 | 16 | 22·6 |
| Larynx | 15 | 10·2 | 4 | 5·0 | 6 | 2·4 | 4 | 6·3 | 2 | 1·4 | 7 | 6·3 |
| Lung | 63 | 30·6 | 10 | 15·0 | 22 | 7·3 | 17 | 18·9 | 4 | 4·2 | 14 | 19·1 |
| Lip | 1 | 2·7 | — | 1·3 | 1 | 0·6 | 1 | 1·6 | — | 0·4 | 3 | 1·7 |
| Tongue | 9 | 5·3 | 3 | 2·6 | 1 | 1·2 | 6 | 3·2 | — | 0·7 | 5 | 3·3 |
| Mouth | 7 | 3·3 | 1 | 1·6 | 1 | 0·6 | 2 | 2·0 | — | 0·4 | 2 | 2·0 |
| Jaw | 4 | 4·2 | 1 | 2·1 | 2 | 0·8 | 3 | 2·6 | — | 0·6 | 1 | 2·6 |
| Pharynx | 8 | 2·9 | — | 1·4 | — | 1·0 | 2 | 1·8 | 1 | 0·4 | 1 | 1·8 |
| Liver | 39 | 29·8 | 17 | 14·6 | 8 | 7·1 | 19 | 18·4 | 2 | 4·0 | 15 | 18·5 |
| Bile passages | 2 | 5·7 | 4 | 2·8 | 3 | 1·4 | 3 | 3·6 | 1 | 0·8 | 3 | 3·6 |
| Pancreas | 10 | 6·4 | 1 | 3·2 | 4 | 1·5 | 2 | 4·0 | 2 | 0·9 | 5 | 4·0 |
| Peritoneum | 3 | 3·1 | — | 1·5 | — | 0·7 | 2 | 1·9 | 1 | 0·4 | 1 | 1·9 |
| Kidney | 6 | 2·7 | 3 | 1·3 | 3 | 0·6 | — | 1·7 | — | 0·4 | 2 | 1·7 |
| Bladder | 14 | 14·1 | 6 | 6·9 | 2 | 3·3 | 13 | 8·7 | 4 | 1·9 | 7 | 8·8 |
| Penis | 2 | 2·5 | 4 | 1·2 | — | 0·6 | 1 | 1·5 | — | 0·3 | 2 | 1·5 |
| Prostate | 42 | 31·7 | 28 | 15·5 | 5 | 7·5 | 27 | 19·6 | 4 | 4·3 | 13 | 19·7 |
| Skin | 3 | 8·0 | 1 | 3·9 | — | 1·9 | 3 | 5·0 | — | 1·1 | 12 | 5·0 |
| Other sites | 19 | 24·3 | 8 | 12·0 | 1 | 5·9 | 12 | 15·1 | 3 | 3·3 | 15 | 15·1 |
| Total | 553 | 553 | 271 | 271 | 131 | 131 | 342 | 342 | 75 | 75 | 344 | 344 |

TABLE XII—(*cont.*).

| *Site of carcinoma.* | *Police.* A. | *Police.* E. | *Diamond cutters.* A. | *Diamond cutters.* E. | *Carmen (Drivers, Stable keepers, Coachmen).* A. | *Carmen (Drivers, Stable keepers, Coachmen).* E. | *Chauffeurs.* A. | *Chauffeurs.* E. | *Firemen and Engine drivers.* A. | *Firemen and Engine drivers.* E. | *Dock labourers.* A. | *Dock labourers.* E. |
|---|---|---|---|---|---|---|---|---|---|---|---|---|
| Oesophagus | 12 | 8·5 | 7 | 7·7 | 16 | 11·5 | 1 | 2·2 | 16 | 16·3 | 8 | 6·3 |
| Stomach | 50 | 57·8 | 44 | 52·2 | 84 | 78·5 | 17 | 15·0 | 120 | 111·2 | 45 | 43·1 |
| Intestines | 5 | 9·5 | 8 | 8·6 | 4 | 13·0 | 3 | 2·5 | 18 | 18·4 | 7 | 7·1 |
| Rectum | 16 | 8·3 | 3 | 7·5 | 14 | 11·4 | 3 | 2·2 | 13 | 16·1 | 6 | 6·2 |
| Larynx | 3 | 2·3 | 2 | 2·1 | 5 | 3·2 | — | 0·6 | 8 | 4·5 | 3 | 1·7 |
| Lung | 10 | 7·0 | 15 | 6·4 | 4 | 9·6 | 4 | 1·8 | 12 | 13·6 | 1 | 5·3 |
| Lip | 3 | 0·6 | — | 0·5 | 2 | 0·8 | — | 0·2 | — | 1·2 | 1 | 0·5 |
| Tongue | 1 | 1·2 | 3 | 1·1 | — | 1·6 | — | 0·3 | 3 | 2·3 | 3 | 0·9 |
| Mouth | — | 0·7 | 1 | 0·7 | 2 | 1·0 | 1 | 0·2 | 1 | 1·4 | 1 | 0·6 |
| Jaw | 1 | 1·0 | — | 0·9 | — | 1·3 | — | 0·2 | — | 1·9 | — | 0·7 |
| Pharynx | — | 0·7 | 2 | 0·6 | 3 | 0·9 | — | 0·2 | — | 1·3 | — | 0·5 |
| Liver | 5 | 6·8 | 9 | 6·2 | 12 | 9·3 | — | 1·8 | 12 | 13·2 | 3 | 5·1 |
| Bile passages | 1 | 1·3 | 2 | 1·2 | 2 | 1·8 | — | 0·3 | 2 | 2·5 | 1 | 1·0 |
| Pancreas | 1 | 1·5 | — | 1·3 | 3 | 2·0 | — | 0·3 | 6 | 2·8 | — | 1·1 |
| Peritoneum | — | 0·7 | 1 | 0·6 | 1 | 1·0 | 2 | 0·2 | — | 1·4 | 1 | 0·5 |
| Kidney | — | 0·6 | 2 | 0·6 | — | 0·8 | — | 0·2 | 1 | 1·2 | — | 0·5 |
| Bladder | 2 | 3·2 | 4 | 2·9 | 11 | 4·4 | — | 0·8 | 10 | 6·2 | 1 | 2·4 |
| Penis | 2 | 0·6 | — | 0·5 | 1 | 0·8 | — | 0·1 | — | 1·1 | 2 | 0·4 |
| Prostate | 8 | 7·2 | 8 | 6·6 | 8 | 9·9 | — | 1·9 | 14 | 14·0 | 8 | 5·4 |
| Skin | 1 | 1·8 | — | 1·7 | — | 2·5 | — | 0·5 | 2 | 3·6 | — | 1·4 |
| Other sites | 6 | 5·7 | 4 | 5·1 | 1 | 7·7 | 2 | 1·5 | 7 | 10·8 | 4 | 4·3 |
| Total | 127 | 127 | 115 | 115 | 173 | 173 | 33 | 33 | 245 | 245 | 95 | 95 |

| *Site of carcinoma.* | *Fishermen.* A. | *Fishermen.* E. | *Green-grocers.* A. | *Green-grocers.* E. | *Commercial travellers.* A. | *Commercial travellers.* E. | *Market gardeners and their assistants.* A. | *Market gardeners and their assistants.* E. | *Navvies.* A. | *Navvies.* E. | *Hall porters and Caretakers.* A. | *Hall porters and Caretakers.* E. |
|---|---|---|---|---|---|---|---|---|---|---|---|---|
| Oesophagus | 18 | 11·5 | 8 | 8·8 | 16 | 7·7 | 24 | 18·1 | 10 | 10·6 | 8 | 6·4 |
| Stomach | 76 | 78·0 | 74 | 59·9 | 39 | 52·6 | 140 | 123·4 | 95 | 72·1 | 33 | 43·6 |
| Intestines | 8 | 12·9 | 5 | 9·9 | 9 | 8·7 | 15 | 20·4 | 7 | 11·9 | 8 | 7·2 |
| Rectum | 6 | 11·3 | 6 | 8·7 | 7 | 7·6 | 19 | 17·9 | 10 | 10·4 | 10 | 6·3 |
| Larynx | 3 | 3·2 | 1 | 2·4 | 3 | 2·1 | 6 | 5·0 | 2 | 2·9 | 4 | 1·8 |
| Lung | 7 | 9·5 | 7 | 7·3 | 15 | 6·4 | 7 | 15·1 | 2 | 8·8 | 14 | 5·3 |
| Lip | 1 | 0·8 | — | 0·6 | — | 0·6 | 3 | 1·3 | 2 | 0·8 | 1 | 0·5 |
| Tongue | 2 | 1·6 | — | 1·3 | 1 | 1·1 | 1 | 2·6 | — | 1·5 | 2 | 0·9 |
| Mouth | 2 | 1·0 | — | 0·8 | 4 | 0·7 | — | 1·6 | — | 0·9 | 2 | 0·6 |
| Jaw | 4 | 1·2 | — | 1·0 | — | 0·9 | — | 2·1 | 1 | 1·2 | — | 0·7 |
| Pharynx | — | 0·9 | 2 | 0·7 | 1 | 0·6 | 1 | 1·4 | — | 0·8 | — | 0·5 |
| Liver | 11 | 9·3 | 10 | 7·1 | 5 | 6·1 | 12 | 14·6 | 12 | 8·6 | 3 | 5·1 |
| Bile passages | 1 | 1·8 | — | 1·4 | — | 1·2 | 4 | 2·8 | 1 | 1·7 | 1 | 1·0 |
| Pancreas | 2 | 2·0 | 2 | 1·5 | 4 | 1·3 | 3 | 3·2 | 2 | 1·8 | — | 1·1 |
| Peritoneum | 3 | 1·0 | — | 0·7 | 2 | 0·7 | 1 | 1·5 | — | 0·9 | 1 | 0·5 |
| Kidney | 2 | 0·8 | 2 | 0·6 | 2 | 0·6 | — | 1·3 | 1 | 0·8 | — | 0·5 |
| Bladder | 3 | 4·4 | 3 | 3·4 | 1 | 3·0 | 8 | 6·9 | 2 | 4·1 | 2 | 2·5 |
| Penis | 1 | 0·8 | — | 0·6 | — | 0·5 | — | 1·2 | — | 0·7 | — | 0·4 |
| Prostate | 13 | 9·9 | 6 | 7·6 | 3 | 6·6 | 19 | 15·6 | 3 | 9·1 | 5 | 5·5 |
| Skin | 5 | 2·5 | 3 | 1·9 | — | 1·7 | 5 | 4·0 | 2 | 2·3 | — | 1·4 |
| Other sites | 4 | 7·6 | 3 | 5·8 | 4 | 5·3 | 4 | 12·0 | 7 | 7·1 | 2 | 4·2 |
| Total | 172 | 172 | 132 | 132 | 116 | 116 | 272 | 272 | 159 | 159 | 96 | 96 |

TABLE XII—(*cont.*).

| *Site of carcinoma.* | *Railway and Tram men.* A. | E. | *Cigar makers and Cigar manufacturers.* A. | E. | *Tobacconists.* A. | E. | *Plumbers.* A. | E. | *Printers.* A. | E. | *Grocers.* A. | E. |
|---|---|---|---|---|---|---|---|---|---|---|---|---|
| Oesophagus | 29 | 30·9 | 8 | 11·0 | 3 | 3·7 | 4 | 3·2 | 5 | 6·7 | 12 | 13·8 |
| Stomach | 211 | 210·5 | 74 | 74·9 | 26 | 25·4 | 14 | 21·8 | 34 | 45·4 | 86 | 93·9 |
| Intestines | 31 | 34·8 | 12 | 12·4 | 2 | 4·2 | 2 | 3·6 | 12 | 7·5 | 14 | 15·5 |
| Rectum | 34 | 30·5 | 7 | 10·8 | 5 | 3·7 | 4 | 3·2 | 10 | 6·6 | 12 | 13·6 |
| Larynx | 6 | 8·5 | 2 | 3·0 | 1 | 1·0 | 2 | 0·9 | 2 | 1·8 | 6 | 3·8 |
| Lung | 29 | 25·7 | 14 | 9·1 | 10 | 3·1 | 2 | 2·7 | 3 | 5·5 | 19 | 11·5 |
| Lip | 6 | 2·2 | 1 | 0·8 | — | 0·3 | — | 0·2 | — | 0·5 | 2 | 1·0 |
| Tongue | 7 | 4·4 | 1 | 1·6 | 3 | 0·5 | 1 | 0·5 | 2 | 1·0 | 1 | 2·0 |
| Mouth | 2 | 2·7 | 1 | 1·0 | — | 0·3 | — | 0·3 | — | 0·6 | — | 1·2 |
| Jaw | 8 | 3·5 | — | 1·2 | 1 | 0·4 | — | 0·4 | — | 0·8 | 3 | 1·6 |
| Pharynx | 3 | 2·5 | 1 | 0·9 | 1 | 0·3 | — | 0·3 | — | 0·5 | 1 | 1·1 |
| Liver | 23 | 25·0 | 10 | 8·9 | 1 | 3·0 | 5 | 2·6 | 7 | 5·4 | 13 | 11·1 |
| Bile passages | 4 | 4·8 | 1 | 1·7 | — | 0·6 | — | 0·5 | 3 | 1·0 | 5 | 2·2 |
| Pancreas | 7 | 5·4 | 2 | 1·9 | 1 | 0·6 | 3 | 0·5 | 2 | 1·2 | 2 | 2·4 |
| Peritoneum | 3 | 2·6 | 1 | 0·9 | — | 0·3 | — | 0·3 | — | 0·6 | — | 1·2 |
| Kidney | 2 | 2·3 | 2 | 0·8 | — | 0·3 | 2 | 0·2 | 2 | 0·5 | — | 1·0 |
| Bladder | 14 | 11·8 | 5 | 4·2 | — | 1·4 | 3 | 1·2 | 5 | 2·5 | 9 | 5·3 |
| Penis | 3 | 2·1 | 1 | 0·7 | — | 0·3 | 1 | 0·2 | — | 0·4 | 1 | 0·9 |
| Prostate | 24 | 26·6 | 12 | 9·4 | 1 | 3·2 | 4 | 2·7 | 7 | 5·7 | 6 | 11·9 |
| Skin | 1 | 6·7 | 2 | 2·4 | — | 0·8 | — | 0·7 | 2 | 1·4 | 4 | 3·0 |
| Other sites | 17 | 20·5 | 8 | 7·4 | 1 | 2·6 | 1 | 2·0 | 4 | 4·4 | 11 | 9·0 |
| Total | 464 | 464 | 165 | 165 | 56 | 56 | 48 | 48 | 100 | 100 | 207 | 207 |

TABLE XIIIa.—*Recorded and Expected Deaths from Carcinoma amongst Wives of Men in Various Trades and Professions.*

| *Site of carcinoma.* | *Publicans' and Hotel keepers' wives.* A. | E. | *Head-teachers' and Teachers' wives.* A. | E. | *Butchers' wives.* A. | E. | *Carpenters' wives.* A. | E. | *Bakers' and Confectioners' wives.* A. | E. | *Smiths' wives.* A. | E. |
|---|---|---|---|---|---|---|---|---|---|---|---|---|
| Oesophagus | — | 2·4 | — | 1·1 | 1 | 1·4 | 4 | 4·9 | 2 | 2·6 | 1 | 1·2 |
| Stomach | 29 | 34·8 | 11 | 16·4 | 18 | 20·6 | 60 | 72·5 | 39 | 37·5 | 17 | 17·4 |
| Intestines | 10 | 11·2 | 5 | 5·2 | 7 | 6·6 | 21 | 23·3 | 13 | 12·0 | 6 | 5·6 |
| Rectum | 6 | 5·8 | 2 | 2·7 | 3 | 3·4 | 16 | 12·1 | 7 | 6·2 | 2 | 2·9 |
| Larynx | 1 | 0·4 | — | 0·2 | — | 0·2 | — | 0·8 | 1 | 0·4 | 1 | 0·2 |
| Lung | 1 | 2·1 | — | 1·0 | 2 | 1·2 | 2 | 4·4 | 2 | 2·3 | 4 | 1·1 |
| Tongue | — | 0·3 | — | 0·1 | — | 0·2 | — | 0·6 | — | 0·3 | — | 0·1 |
| Mouth | — | 0·5 | — | 0·2 | — | 0·3 | 1 | 1·0 | — | 0·5 | — | 0·3 |
| Jaw | 1 | 0·5 | — | 0·2 | — | 0·3 | 4 | 1·1 | — | 0·5 | — | 0·2 |
| Pharynx | — | 0·2 | — | 0·1 | — | 0·1 | 1 | 0·4 | — | 0·2 | 1 | 0·1 |
| Liver | 8 | 11·3 | 4 | 5·3 | 3 | 6·7 | 22 | 23·5 | 10 | 12·1 | 8 | 5·6 |
| Bile passages | 8 | 5·6 | — | 2·6 | — | 3·3 | 14 | 11·8 | 6 | 6·1 | 2 | 2·8 |
| Pancreas | 2 | 1·6 | — | 0·7 | 1 | 0·9 | 3 | 3·3 | 3 | 1·7 | — | 0·8 |
| Peritoneum | — | 1·8 | 1 | 0·9 | 1 | 1·1 | 3 | 3·8 | 4 | 2·0 | — | 0·9 |
| Kidney | — | 0·3 | — | 0·2 | 1 | 0·2 | — | 0·7 | — | 0·4 | — | 0·2 |
| Bladder | — | 1·2 | — | 0·6 | — | 0·7 | 1 | 2·6 | — | 1·3 | 1 | 0·6 |
| Corpus uteri | 8 | 5·4 | 3 | 2·5 | 3 | 3·2 | 10 | 11·1 | 2 | 5·8 | 1 | 2·7 |
| Cervix uteri | 14 | 7·7 | 3 | 3·6 | 11 | 4·6 | 19 | 16·0 | 7 | 8·3 | 5 | 3·8 |
| Uterus n.o.s. | 5 | 2·5 | 2 | 1·2 | 2 | 1·5 | 8 | 5·2 | 4 | 2·7 | — | 1·2 |
| Vagina and vulva | 1 | 1·2 | — | 0·6 | 1 | 0·7 | 4 | 2·6 | 1 | 1·3 | 2 | 0·6 |
| Skin | 1 | 1·0 | — | 0·5 | 1 | 0·6 | 1 | 2·1 | 2 | 1·1 | — | 0·5 |
| Breast | 34 | 22·2 | 24 | 10·4 | 15 | 13·1 | 53 | 46·2 | 25 | 23·9 | 12 | 11·1 |
| Ovary | — | 5·5 | 4 | 2·6 | 7 | 3·3 | 13 | 11·5 | 7 | 5·9 | 1 | 2·8 |
| Other sites | 1 | 4·6 | 2 | 2·2 | — | 2·8 | 9 | 9·5 | 5 | 4·9 | 1 | 2·3 |
| Total | 130 | 130 | 61 | 61 | 77 | 77 | 271 | 271 | 140 | 140 | 65 | 65 |

*Note.*—A. = Number of deaths actually recorded.
E. = Number of deaths expected from the site distribution of carcinoma deaths amongst all married women.

TABLE I—(*cont.*).

| *Site of carcinoma.* | *Shoe makers' wives.* A. | E. | *Farmers' wives.* A. | E. | *Farm labourers' wives.* A. | E. | *Stock farmers' wives.* A. | E. | *House painters' wives.* A. | E. | *Weavers' wives.* A. | E. |
|---|---|---|---|---|---|---|---|---|---|---|---|---|
| Oesophagus | 1 | 2·8 | 27 | 21·4 | 16 | 7·7 | 6 | 3·9 | 2 | 2·4 | 2 | 1·3 |
| Stomach | 56 | 41·0 | 370 | 316·7 | 176 | 113·5 | 61 | 58·1 | 39 | 35·6 | 26 | 19·3 |
| Intestines | 9 | 13·2 | 107 | 101·7 | 22 | 36·5 | 25 | 18·7 | 10 | 11·4 | 10 | 6·2 |
| Rectum | 9 | 6·8 | 45 | 52·6 | 15 | 18·9 | 8 | 9·7 | 2 | 5·9 | 2 | 3·2 |
| Larynx | — | 0·5 | 5 | 3·7 | — | 1·3 | 1 | 0·7 | — | 0·4 | — | 0·2 |
| Lung | 3 | 2·5 | 14 | 19·3 | 2 | 6·9 | 3 | 3·5 | 1 | 2·2 | 2 | 1·2 |
| Tongue | — | 0·3 | 1 | 2·6 | 2 | 0·9 | 2 | 0·5 | — | 0·3 | — | 0·2 |
| Mouth | — | 0·6 | 1 | 4·6 | — | 1·7 | — | 0·8 | — | 0·5 | — | 0·3 |
| Jaw | — | 0·6 | 4 | 4·4 | 2 | 1·6 | 1 | 0·8 | — | 0·5 | — | 0·3 |
| Pharynx | — | 0·2 | 1 | 1·8 | — | 0·6 | 1 | 0·3 | — | 0·2 | — | 0·1 |
| Liver | 8 | 13·3 | 166 | 102.6 | 47 | 36·8 | 17 | 18·8 | 10 | 11·5 | 11 | 6·2 |
| Bile passages | 8 | 6·6 | 64 | 51·3 | 14 | 18·4 | 11 | 9·4 | 6 | 5·8 | 3 | 3·1 |
| Pancreas | 1 | 1·9 | 8 | 14·4 | 6 | 5·2 | 3 | 2·6 | — | 1·6 | 1 | 0·9 |
| Peritoneum | 5 | 2·2 | 10 | 16·7 | 1 | 6·0 | 4 | 3·1 | 2 | 1·9 | 1 | 1·1 |
| Kidney | — | 0·4 | 2 | 3·0 | 2 | 1·1 | 1 | 0·5 | 1 | 0·3 | — | 0·2 |
| Bladder | 1 | 1·5 | 3 | 11·2 | 3 | 4·0 | 1 | 2·1 | — | 1·3 | — | 0·7 |
| Corpus uteri | 5 | 6·3 | 40 | 48·7 | 8 | 17·5 | 6 | 8·9 | 2 | 5·5 | 2 | 3·0 |
| Cervix uteri | 5 | 9·1 | 20 | 70·0 | 8 | 25·1 | 4 | 12·8 | 14 | 7·9 | 2 | 4·3 |
| Uterus n.o.s. | — | 2·9 | 12 | 22·6 | 7 | 8·1 | 2 | 4·1 | 1 | 2·5 | 1 | 1·4 |
| Vagina and vulva | 1 | 1·5 | 5 | 11·4 | — | 4·1 | 1 | 2·1 | 3 | 1·3 | 1 | 0·7 |
| Skin | 2 | 1·2 | 10 | 9·0 | 3 | 3·2 | — | 1·6 | — | 1·0 | — | 0·5 |
| Breast | 26 | 26·1 | 181 | 201·8 | 67 | 72·3 | 41 | 37·0 | 33 | 22·7 | 4 | 12·2 |
| Ovary | 7 | 6·5 | 43 | 50·3 | 9 | 18·0 | 18 | 9·2 | 3 | 5·7 | 2 | 3·1 |
| Other sites | 6 | 5·0 | 44 | 41·2 | 14 | 14·6 | — | 7·8 | 4 | 4·6 | 2 | 2·3 |
| Total | 153 | 153 | 1183 | 1183 | 424 | 424 | 217 | 217 | 133 | 133 | 72 | 72 |

| *Site of carcinoma.* | *Wives of University men.* A. | E. | *General Labourers' wives.* A. | E. | *Wives of Office staffs.* A. | E. | *Tailors' wives.* A. | E. | *Masons' wives.* A. | E. | *Wives of Dealers in fuel.* A. | E. |
|---|---|---|---|---|---|---|---|---|---|---|---|---|
| Oesophagus | — | 1·3 | 7 | 8·1 | 1 | 3·4 | 5 | 2·3 | — | 2·4 | 2 | 1·1 |
| Stomach | 7 | 18·7 | 121 | 120·2 | 20 | 54·4 | 31 | 34·3 | 40 | 35·1 | 14 | 16·1 |
| Intestines | 10 | 6·0 | 38 | 38·6 | 28 | 17·3 | 7 | 11·0 | 14 | 11·3 | 5 | 5·2 |
| Rectum | 4 | 3·1 | 22 | 20·0 | 7 | 8·9 | 6 | 5·7 | 11 | 5·8 | 2 | 2·7 |
| Larynx | — | 0·2 | 2 | 1·4 | 1 | 0·6 | — | 0·4 | — | 0·4 | 2 | 0·2 |
| Lung | — | 1·1 | 7 | 7·3 | 4 | 3·3 | 1 | 2·1 | — | 2·1 | 1 | 1·0 |
| Tongue | — | 0·2 | — | 1·0 | — | 0·4 | 1 | 0·3 | — | 0·3 | — | 0·1 |
| Mouth | — | 0·3 | — | 1·8 | — | 0·8 | — | 0·5 | — | 0·5 | — | 0·2 |
| Jaw | — | 0·3 | 1 | 1·7 | — | 0·7 | 1 | 0·5 | — | 0·5 | 1 | 0·2 |
| Pharynx | — | 0·1 | — | 0·7 | — | 0·3 | — | 0·2 | — | 0·2 | — | 0·1 |
| Liver | 1 | 6·1 | 37 | 38·9 | 10 | 17·4 | 8 | 11·1 | 6 | 11·4 | — | 5·2 |
| Bile passages | 2 | 3·0 | 23 | 19·5 | 4 | 8·7 | 8 | 5·6 | 6 | 5·7 | 6 | 2·6 |
| Pancreas | — | 0·9 | 3 | 5·5 | 4 | 2·5 | 1 | 1·6 | — | 1·6 | 1 | 0·7 |
| Peritoneum | 2 | 1·0 | 7 | 6·3 | 4 | 2·8 | 4 | 1·8 | 2 | 1·8 | — | 0·8 |
| Kidney | — | 0·2 | — | 1·1 | 2 | 0·5 | — | 0·3 | — | 0·3 | — | 0·1 |
| Bladder | 1 | 0·7 | 3 | 4·3 | 1 | 1·9 | 2 | 1·2 | 2 | 1·2 | 1 | 0·6 |
| Corpus uteri | 5 | 2·9 | 15 | 18·5 | 8 | 8·3 | 5 | 5·3 | 3 | 5·4 | 2 | 2·5 |
| Cervix uteri | 2 | 4·1 | 35 | 26·6 | 10 | 11·9 | 5 | 7·6 | 7 | 7·8 | 7 | 3·6 |
| Uterus n.o.s. | 2 | 1·3 | 12 | 8·6 | 5 | 3·8 | 5 | 2·4 | 3 | 2·5 | 3 | 1·1 |
| Vagina and vulva | — | 0·7 | 2 | 4·3 | 3 | 1·9 | 2 | 1·2 | — | 1·3 | 1 | 0·6 |
| Skin | — | 0·5 | 8 | 3·4 | 1 | 1·5 | 1 | 1·0 | 2 | 1·0 | 1 | 0·5 |
| Breast | 18 | 11·9 | 75 | 76·6 | 58 | 34·3 | 26 | 21·8 | 25 | 22·3 | 5 | 10·2 |
| Ovary | 11 | 3·0 | 18 | 19·0 | 27 | 8·5 | 7 | 5·4 | 6 | 5·6 | 3 | 2·5 |
| Other sites | 5 | 2·4 | 13 | 15·6 | 3 | 6·9 | 2 | 4·4 | 4 | 4·5 | 3 | 2·1 |
| Total | 70 | 70 | 449 | 449 | 201 | 201 | 128 | 128 | 131 | 131 | 60 | 60 |

### TABLE XIIIa—(*cont.*).

| *Site of carcinoma.* | *Wives of Bargemen* | | *Police wives.* | | *Wives of Diamond cutters.* | | *Carmen's wives.* | | *Chauffeurs' wives.* | | *Wives of firemen.* | |
|---|---|---|---|---|---|---|---|---|---|---|---|---|
| | A. | E. | A. | E. | A. | E. | A. | E. | A. | E. | A. | E. |
| Oesophagus | 4 | 2·0 | — | 0·7 | — | 0·9 | 1 | 0·7 | 1 | 0·7 | — | 1·9 |
| Stomach | 17 | 30·2 | 8 | 10·7 | 14 | 12·6 | 13 | 9·6 | 7 | 9·6 | 16 | 28·1 |
| Intestines | 9 | 9·7 | 3 | 3·4 | 4 | 4·0 | 3 | 3·1 | 3 | 3·1 | 8 | 9·0 |
| Rectum | 5 | 5·0 | 1 | 1·8 | 2 | 2·1 | — | 1·6 | 1 | 1·6 | 3 | 4·7 |
| Larynx | — | 0·4 | — | 0·1 | — | 0·1 | — | 0·1 | — | 0·1 | 2 | 0·3 |
| Lung | 3 | 1·8 | — | 0·7 | 1 | 0·8 | 1 | 0·6 | 1 | 0·6 | 1 | 1·7 |
| Tongue | 2 | 0·2 | — | 0·1 | — | 0·1 | — | 0·1 | 1 | 0·1 | — | 0·2 |
| Mouth | 1 | 0·4 | — | 0·2 | — | 0·2 | — | 0·1 | — | 0·1 | — | 0·4 |
| Jaw | — | 0·4 | 1 | 0·1 | — | 0·2 | — | 0·1 | — | 0·1 | 1 | 0·4 |
| Pharynx | — | 0·2 | — | 0·1 | — | 0·1 | 1 | 0·1 | — | 0·1 | 9 | 9·1 |
| Liver | 8 | 9·8 | — | 3·5 | 5 | 4·1 | 3 | 3·1 | — | 3·1 | — | 0·2 |
| Bile passages | 6 | 4·9 | 1 | 1·7 | 2 | 2·0 | 2 | 1·6 | — | 1·6 | 6 | 4·6 |
| Pancreas | 2 | 1·4 | 1 | 0·5 | — | 0·6 | — | 0·4 | 1 | 0·4 | 1 | 1·3 |
| Peritoneum | 2 | 1·6 | — | 0·6 | — | 0·7 | — | 0·5 | 1 | 0·5 | 3 | 1·5 |
| Kidney | 1 | 0·3 | — | 0·1 | 2 | 0·1 | — | 0·1 | — | 0·1 | — | 0·3 |
| Bladder | — | 1·1 | — | 0·4 | — | 0·4 | 2 | 0·3 | — | 0·3 | — | 1·0 |
| Corpus uteri | 3 | 4·7 | — | 1·6 | 2 | 1·9 | 2 | 1·5 | 2 | 1·5 | 3 | 4·3 |
| Cervix uteri | 14 | 6·7 | 12 | 2·4 | 2 | 2·8 | 1 | 2·1 | 5 | 2·1 | 12 | 6·2 |
| Uterus n.o.s. | 3 | 2·2 | 1 | 0·8 | 1 | 0·9 | — | 0·7 | 1 | 0·7 | 7 | 2·0 |
| Vagina and vulva | 1 | 1·1 | — | 0·4 | 1 | 0·4 | — | 0·3 | — | 0·3 | — | 1·0 |
| Skin | — | 0·9 | — | 0·3 | — | 0·4 | 1 | 0·3 | 2 | 0·3 | 1 | 0·8 |
| Breast | 19 | 19·3 | 9 | 6·8 | 6 | 8·0 | 4 | 6·1 | 6 | 6·1 | 24 | 17·9 |
| Ovary | 6 | 4·8 | 3 | 1·7 | 5 | 2·0 | — | 1·5 | 1 | 1·5 | 3 | 4·5 |
| Other sites | 7 | 3·9 | — | 1·3 | — | 1·6 | 2 | 1·4 | 3 | 1·4 | 5 | 3·6 |
| Total | 113 | 113 | 40 | 40 | 47 | 47 | 36 | 36 | 36 | 36 | 105 | 105 |

| *Site of carinoma.* | *Wives of fishermen.* | | *Wives of greengrocers.* | | *Wives of Commercial travellers.* | | *Wives of Market gardeners.* | | *Wives of Navvies.* | | *Wives of Hall porters.* | |
|---|---|---|---|---|---|---|---|---|---|---|---|---|
| | A. | E. | A. | E. | A. | E. | A. | E. | A. | E. | A. | E. |
| Oesophagus | 3 | 0·9 | — | 0·9 | 1 | 1·3 | 1 | 2·6 | — | 1·4 | — | 0·9 |
| Stomach | 10 | 12·6 | 10 | 13·1 | 16 | 19·0 | 55 | 37·7 | 18 | 20·1 | 5 | 13·9 |
| Intestines | 2 | 4·0 | 3 | 4·2 | 3 | 6·1 | 17 | 12·1 | 2 | 6·4 | 2 | 4·5 |
| Rectum | 2 | 2·1 | 1 | 2·2 | 2 | 3·2 | 7 | 6·3 | 2 | 3·3 | 3 | 2·3 |
| Larynx | — | 0·1 | — | 0·2 | — | 0·2 | — | 0·4 | 1 | 0·2 | 1 | 0·2 |
| Lung | 1 | 0·8 | 1 | 0·8 | 1 | 1·2 | 3 | 2·3 | 3 | 1·2 | 1 | 0·8 |
| Tongue | — | 0·1 | — | 0·1 | — | 0·2 | — | 0·3 | — | 0·2 | — | 0·1 |
| Mouth | — | 0·2 | — | 0·2 | — | 0·3 | — | 0·5 | — | 0·3 | — | 0·2 |
| Jaw | — | 0·2 | — | 0·2 | — | 0·3 | — | 0·5 | 1 | 0·3 | — | 0·2 |
| Pharynx | — | 0·1 | — | 0·1 | — | 0·1 | — | 0·2 | — | 0·1 | — | 0·1 |
| Liver | 5 | 4·1 | 3 | 4·2 | 2 | 6·2 | 8 | 12·2 | 14 | 6·5 | 2 | 4·5 |
| Bile passages | 1 | 2·0 | — | 2·1 | — | 3·1 | 3 | 6·1 | 4 | 3·3 | 2 | 2·3 |
| Pancreas | — | 0·6 | 1 | 0·6 | 2 | 0·9 | 1 | 1·7 | 1 | 0·9 | 1 | 0·6 |
| Peritoneum | 1 | 0·7 | 4 | 0·7 | 1 | 1·0 | 3 | 2·0 | — | 1·1 | — | 0·7 |
| Kidney | — | 0·1 | — | 0·1 | 1 | 0·2 | — | 0·4 | — | 0·2 | — | 0·1 |
| Bladder | — | 0·4 | 2 | 0·5 | — | 0·7 | 3 | 1·3 | 1 | 0·7 | 1 | 0·5 |
| Corpus uteri | 2 | 1·9 | 2 | 2·0 | 3 | 2·9 | 7 | 5·8 | 4 | 3·1 | 3 | 2·1 |
| Cervix uteri | 2 | 2·8 | 7 | 2·9 | 8 | 4·2 | — | 8·3 | 12 | 4·4 | 7 | 3·1 |
| Uterus n.o.s. | — | 0·9 | 1 | 0·9 | — | 1·4 | 2 | 2·7 | 2 | 1·4 | — | 1·0 |
| Vagina and vulva | 1 | 0·5 | 1 | 0·5 | 1 | 0·7 | 1 | 1·4 | 2 | 0·7 | 1 | 0·5 |
| Skin | — | 0·4 | — | 0·4 | — | 0·5 | 1 | 1·1 | — | 0·6 | — | 0·4 |
| Breast | 9 | 8·0 | 12 | 8·4 | 23 | 12·0 | 19 | 24·1 | 7 | 12·8 | 10 | 8·9 |
| Ovary | 6 | 2·0 | — | 2·1 | 4 | 3·0 | 5 | 6·0 | 1 | 3·2 | 7 | 2·2 |
| Other sites | 2 | 1·5 | 1 | 1·6 | 3 | 2·3 | 5 | 5·0 | — | 2·6 | 6 | 1·9 |
| Total | 47 | 47 | 49 | 49 | 71 | 71 | 141 | 141 | 75 | 75 | 52 | 52 |

TABLE XIIIa—(*cont.*).

| *Site of carcinoma.* | *Wives of Railway and Tram men.* A. | E. | *Wives of Grocers.* A. | E. |
|---|---|---|---|---|
| Oesophagus | — | 3·4 | 1 | 1·6 |
| Stomach | 30 | 50·9 | 35 | 23·7 |
| Intestines | 13 | 16·3 | 7 | 7·7 |
| Rectum | 11 | 8·5 | 2 | 4·0 |
| Larynx | 1 | 0·6 | — | 0·3 |
| Lung | 4 | 3·1 | — | 1·5 |
| Tongue | — | 0·4 | — | 0·2 |
| Mouth | — | 0·7 | — | 0·3 |
| Jaw | 3 | 0·7 | — | 0·3 |
| Pharynx | — | 0·3 | — | 0·1 |
| Liver | 11 | 16·5 | 3 | 7·7 |
| Bile passages | 9 | 8·2 | 3 | 3·9 |
| Pancreas | 7 | 2·3 | — | 1·1 |
| Peritoneum | 7 | 2·7 | 1 | 1·3 |
| Kidney | 2 | 0·5 | — | 0·2 |
| Bladder | 2 | 1·8 | — | 0·8 |
| Corpus uteri | 2 | 7·8 | 4 | 3·7 |
| Cervix uteri | 16 | 11·2 | 7 | 5·3 |
| Uterus n.o.s. | 3 | 3·6 | 1 | 1·7 |
| Vagina and vulva | 1 | 1·8 | — | 0·9 |
| Skin | — | 1·4 | — | 0·7 |
| Breast | 42 | 32·5 | 21 | 15·2 |
| Ovary | 20 | 8·1 | 4 | 3·8 |
| Other sites | 6 | 6·7 | — | 3·0 |
| Total | 190 | 190 | 89 | 89 |

TABLE XIIIb.—*Recorded and Expected Deaths from Carcinoma amongst Unmarried Women of Various Occupations.*

| *Site of carcinoma.* | *Teachers.* A. | E. | *Nurses.* A. | E. | *Nuns and Religieuses.* A. | E. | *Domestics (female), unmarried.* A. | E. | *Needle-women, unmarried.* A. | E. |
|---|---|---|---|---|---|---|---|---|---|---|
| Oesophagus | 4 | 1·8 | — | 1·0 | 4 | 3·3 | 8 | 7·3 | — | 2·7 |
| Stomach | 16 | 29·8 | 15 | 16·0 | 60 | 53·4 | 132 | 118·4 | 36 | 42·8 |
| Intestines | 12 | 11·0 | 3 | 5·9 | 19 | 19·8 | 34 | 43·9 | 15 | 15·9 |
| Rectum | 5 | 5·2 | 3 | 2·8 | 4 | 9·3 | 22 | 20·5 | 8 | 7·4 |
| Larynx | — | 0·4 | — | 0·2 | — | 0·7 | 3 | 1·4 | 1 | 0·5 |
| Lung | 2 | 2·2 | 5 | 1·2 | 3 | 4·0 | 11 | 8·9 | 3 | 3·2 |
| Tongue | — | 0·3 | — | 0·2 | 3 | 0·5 | — | 1·2 | 1 | 0·5 |
| Mouth | — | 0·2 | — | 0·1 | — | 0·3 | — | 0·7 | — | 0·2 |
| Jaw | 1 | 0·4 | — | 0·2 | — | 0·6 | 1 | 1·4 | — | 0·5 |
| Pharynx | — | 0·2 | — | 0·1 | — | 0·3 | 1 | 0·8 | — | 0·3 |
| Liver | 5 | 7·9 | 3 | 4·2 | 17 | 14·1 | 27 | 31·2 | 9 | 11·3 |
| Bile-passages | 1 | 1·8 | 2 | 1·0 | 5 | 3·3 | 12 | 7·2 | 2 | 2·6 |
| Pancreas | — | 0·9 | 1 | 0·5 | 2 | 1·6 | 5 | 3·6 | 2 | 1·3 |
| Peritoneum | — | 1·5 | — | 0·8 | 1 | 2·7 | 8 | 5·9 | 4 | 2·1 |
| Kidney | — | 0·3 | — | 0·2 | — | 0·5 | — | 1·2 | — | 0·5 |
| Bladder | 1 | 1·4 | — | 0·7 | 1 | 2·5 | 10 | 5·2 | — | 2·0 |
| Corpus uteri | 7 | 3·6 | 2 | 2·0 | 6 | 6·5 | 7 | 14·5 | 4 | 5·2 |
| Cervix uteri | 1 | 2·6 | 2 | 1·4 | 5 | 4·6 | 9 | 10·2 | 6 | 3·7 |
| Uterus n.o.s. | 6 | 2·6 | 1 | 1·4 | 3 | 4·6 | 5 | 10·2 | 1 | 3·7 |
| Vagina and vulva | — | 0·8 | 1 | 0·4 | — | 1·5 | 1 | 3·3 | — | 1·1 |
| Skin | 1 | 1·2 | — | 0·6 | — | 2·2 | 4 | 4·9 | — | 1·8 |
| Breast | 34 | 24·1 | 13 | 12·9 | 44 | 43·1 | 95 | 95·6 | 34 | 34·6 |
| Ovary | 8 | 6·1 | 8 | 3·3 | 11 | 10·9 | 29 | 24·3 | 16 | 8·8 |
| Other sites | 6 | 3·8 | — | 1·9 | 9 | 6·7 | 13 | 15·2 | 6 | 5·3 |
| Total | 110 | 110 | 59 | 59 | 197 | 197 | 437 | 437 | 158 | 158 |

*Note.*—A. = Number of deaths actually recorded.
E. = Number of deaths expected from the site distribution of carcinoma deaths amongst all single women.

The total mortality from carcinoma for the different trades and professions, from age-classes 40–49 and 50–59 only, is shown in Table XIV as percentages of the number to be expected.

TABLE XIV.—*Total Mortality as Percentages of Expected Number.*

| | Per cent. | | Per cent. |
|---|---|---|---|
| Publicans, etc. | 200 | Diamond-cutters | 100 |
| Headteachers | 91 | Firemen | 61 |
| Butchers | 161 | Fishermen | 194 |
| Bakers | 102 | Greengrocers | 220 |
| Smiths | 69 | Navvies | 62 |
| Shoemakers | 100 | Hall-porters | 100 |
| Farmers | 92 | Cigar-manufacturers and makers | 155 |
| House-painters | 100 | Tobacconists | 121 |
| Weavers | 140 | Domestics (females) | 72 |
| University-men | 100 | Needlewomen | 129 |
| Office-staffs | 97 | Plumbers | 225 |
| Tailors | 100 | Printers | 117 |
| Masons | 91 | Grocers | 106 |
| Bargemen | 90 | Police | 79 |

It is quite evident that there is not an equal tendency among all people to be affected by carcinoma, and this shows itself in a distribution by organs typical for each trade. The total mortality is the sum of the carcinoma mortalities of all organs.

In those trades in which the total mortality is 100 per cent—equal to that of the average population—a relatively increased frequency for a particular organ also means an absolute increase in the carcinoma frequency for that organ. But in those trades in which the total mortality is much higher or lower than 100 per cent, a relative increase or decrease need not necessarily mean a higher or lower absolute carcinoma frequency. In these cases the second method, working from the age-classes 40–49 and 50–59, has a correcting influence.

For instance, whereas we expected 180 cases of carcinoma of the stomach among publicans, etc., we actually found only 119. This would seem to be a reduced incidence, but if we look at the numbers for the age-groups 40–49 and 50–59 we find that the incidence is actually increased—37 instead of the expected 30.

In those trades where the total frequency differs markedly from that of the general population we should calculate the number of carcinomas to be expected in each organ, not by distributing the number of cases found for the trade over the different organs according to the average distribution for the general population, but by distributing $\frac{100}{x}$ times that number found over the different organs, where $x$ is the total mortality for the trade expressed as a percentage of the number to be expected as shown in Table XIV.

Thus we have found that for publicans, etc., the total mortality from carcinoma is 200 per cent that of the general population. If in accordance with the proportions in the general population we distributed the actual number of cases of carcinoma over the various organs, we should expect 180 cases of carcinoma of the stomach. But we have distributed double the number of cases that would have been expected on the basis of the general population. Accordingly we should not compare the 119 cases found with 180, but only with 90, which is the number of carcinomas of the stomach to be expected among publicans at ages between 40 and 60 if they had the same frequency as in the average population.

Working in accordance with both these methods we get the results shown in Table XV, and by comparing the figures for the men and their wives we can tell whether the excess found for an organ depends on the occupation or on the environment.

TABLE XV.—*Organs Affected by Carcinoma in Excess or Defect of Expectation in Different Occupations.*

| *Occupation group.* | *Increased.** | *Probably† increased.* | *Probably† reduced.* | *Reduced.** | *Comments.* |
|---|---|---|---|---|---|
| Farmers | stomach<br>skin | lip | .. | intestines<br>larynx<br>lung<br>tongue<br>pancreas | Farmers' wives also have stomach excess. |
| Agricultural labourers | stomach<br>skin | .. | pancreas<br>kidney<br>tongue | rectum<br>larynx<br>lung | Among farmers are many who are no more than agricultural labourers. |
| Stock farmers | stomach | .. | intestines | bladder | .. |
| Market gardeners | .. | .. | lung | .. | .. |
| Greengrocers | stomach | liver | .. | .. | .. |
| Fishermen | oesophagus<br>stomach<br>jaw | liver<br>skin<br>peritoneum | .. | .. | In Holland the fishermen's wives mend the nets, consequently no such excess of lip carcinoma as in England where the needles are put in the mouths. |
| University men | lung<br>pharynx<br>prostate | tongue | .. | stomach | Clergymen have much prostate and physicians much lung carcinoma. |
| Masters (Grammar-schools, etc.) | lung<br>prostate | intestines<br>penis | .. | stomach | .. |
| Teachers | intestines | liver | .. | stomach | Lady teachers show no increase for intestine. |
| Office staffs | lung<br>intestines | mouth<br>pharynx | .. | stomach | Are always in smoky environment. |
| Publicans, Hotel keepers, Waiters, Coffeehouse keepers | oesophagus<br>larynx<br>lung<br>tongue<br>mouth<br>pharynx<br>bladder | stomach<br>intestines<br>rectum<br>liver<br>pancreas<br>prostate | .. | .. | Is this an indication of the possibility of a general disposition for carcinoma? |
| Soldiers | larynx | tongue<br>liver<br>prostate | .. | stomach | The Indian soldiers have no increased liver-frequency. Dutch land-forces and English Navy quite agree. |

* i.e. by three or more times the standard error.

† "Probably" means: the increase or reduction is twice, but not three times, the standard error.

TABLE XV—(*cont.*).

| *Occupation group.* | *Increased.** | *Probably† increased.* | *Probably† reduced.* | *Reduced.** | *Comments.* |
|---|---|---|---|---|---|
| Railwaymen, etc. | .. | lip<br>jaw | skin | .. | .. |
| Firemen | .. | .. | oesophagus<br>intestines<br>rectum | stomach | .. |
| Smiths | penis | jaw | lung<br>oesophagus<br>liver | stomach | .. |
| Commercial travellers | lung<br>mouth | oesophagus<br>pancreas | .. | .. | .. |
| Butchers | lung<br>bladder | stomach<br>intestines<br>prostate | .. | .. | Any connection between lung and echinococcus? In my opinion wrong diagnosis is not probable ; liver carcinoma is not increased either. |
| Tailors | prostate | penis | .. | .. | .. |
| Drapers | lung | rectum<br>larynx<br>kidney | .. | stomach | .. |
| Carpenters | .. | penis | .. | .. | .. |
| Masons | .. | .. | .. | .. | .. |
| Grocers | .. | lung | .. | .. | .. |
| Shoemakers | .. | mouth<br>bladder | .. | .. | Skin : not increased. Is bladder due to aniline? |
| Diamond cutters | lung | .. | .. | .. | .. |
| Police | .. | rectum<br>lip | stomach | .. | .. |
| Hall porters | lung | mouth | .. | .. | Smoke in closed space. |
| Cigar makers and cigar manufacturers | stomach<br>lung | prostate | .. | .. | .. |
| Tobacconists | lung | tongue | .. | .. | Smoke in closed space. |
| Plumbers | .. | liver<br>pancreas | .. | .. | Lead ? |
| Printers | .. | intestines | .. | .. | Lead ? |
| Weavers | stomach<br>liver | .. | .. | .. | Is liver-carcinoma a trade illness? See Weavers' wives. |
| Carmen | bladder | pharynx | intestines | .. | .. |
| Dock labourers | .. | tongue | .. | .. | .. |
| Navvies | .. | .. | intestines | lung<br>prostate | *Oil venders* are only a small group but have an excessively high stomach frequency (26 of 35 carcinomas). |
| Bargemen | .. | skin | .. | .. | |
| General labourers | oesophagus | penis | .. | lung | |
| Miners | stomach | .. | .. | prostate | |

*Wives of—*

| | | | | | |
|---|---|---|---|---|---|
| Farmers | stomach<br>liver | .. | bladder<br>uterus | cervix uteri | .. |
| Farmers' labourers | oesophagus<br>stomach | .. | intestines<br>corpus uteri<br>ovary | cervix uteri | Despite many children, few cervix carcinomas, so cervix rupture is of secondary importance. |
| Stock farmers | .. | ovary | cervix uteri | .. | .. |

TABLE XV—(*cont.*).

| *Occupation group.* | *Increased.** | *Probably† increased.* | *Probably† reduced.* | *Reduced.** | *Comments.* |
|---|---|---|---|---|---|
| | | | *Wives of—* | | |
| Market gardeners | .. | stomach | cervix uteri | .. | .. |
| Greengrocers | .. | cervix uteri | .. | .. | .. |
| Fishermen | .. | ovary | .. | .. | .. |
| University men | ovary | .. | liver | stomach | .. |
| Teachers | breast | .. | .. | .. | .. |
| Office staffs | breast ovary | intestines | .. | stomach | .. |
| Publicans | .. | cervix uteri breast | ovary | .. | .. |
| Railwaymen | ovary | peritoneum pancreas | stomach | .. | .. |
| Firemen | .. | cervix uteri | stomach | .. | .. |
| Commercial travellers | breast | cervix uteri | .. | .. | .. |
| Butchers | cervix uteri | .. | .. | .. | .. |
| Masons | .. | rectum | .. | .. | .. |
| Dealers in fuel | .. | .. | liver | .. | .. |
| House painters | .. | cervix uteri breast | .. | .. | .. |
| Shoemakers | .. | stomach | .. | .. | .. |
| Police | cervix uteri | .. | .. | .. | .. |
| Hall porters | ovary | cervix uteri | stomach | .. | .. |
| Weavers | .. | liver | breast | .. | .. |
| Navvies | cervix uteri | liver | .. | .. | .. |
| Bargemen | tongue cervix uteri | .. | stomach | .. | .. |
| General labourers | .. | skin | .. | .. | .. |
| Lady teachers | .. | breast | stomach | .. | .. |
| Nurses | lung | ovary | .. | .. | Is the lung carcinoma dependent on ether or on a better diagnosis? |
| Domestics (females) | .. | .. | liver uterus (corpus and unspecified) breast | intestines | .. |
| Needlewomen | ovary | .. | .. | .. | .. |

When arranged according to the *organs*, we find excess or deficiency of carcinoma for the following groups of men :

| *Organ.* | *Increased.* | *Probably increased.* | *Probably reduced.* | *Reduced.* |
|---|---|---|---|---|
| Oesophagus | Barmen<br>General labourers<br>Fishermen | Commercial travellers | Firemen<br>Smiths | .. |
| Stomach | Fishermen<br>Weavers<br>Farmers<br>Agricultural lab.<br>Stock farmers<br>St. Farmhands<br>Greengrocers<br>Cigar makers<br>Miners | Barmen<br>Butchers | Police | Firemen<br>Drapers<br>Smiths.<br>Soldiers.<br>University men.<br>Masters.<br>Office staffs.<br>Teachers |

TABLE XV—(*cont.*).

| *Organ.* | *Increased.* | *Probably increased.* | *Probably reduced.* | *Reduced.* |
|---|---|---|---|---|
| Lung | Drapers<br>Barmen<br>Butchers<br>University men<br>Masters<br>Office staffs<br>Diamond cutters<br>Comm. travellers<br>Hall porters<br>Cigar makers<br>Tobacconists | Grocers | Smiths<br>Market gardeners | Farmers.<br>Agricultural lab.<br>General lab.<br>Navvies. |
| Larynx | Barmen<br>Soldiers | Drapers | .. | Farmers.<br>Agricultural lab. |
| Intestines | Teachers<br>Office staffs | Barmen<br>Butchers<br>Masters<br>Printers | Carmen<br>Firemen<br>Stock farmers<br>Navvies | Farmers. |
| Rectum | .. | Barmen<br>Drapers<br>Police | Firemen | Agricultural lab. |
| Bladder | Butchers<br>Barmen<br>Car men | Shoemakers | .. | Stock farmers. |
| Prostate | University men<br>Masters<br>Tailors | Barmen<br>Butchers<br>Soldiers<br>Cigar makers | ..<br>.. | Navvies.<br>Miners. |
| Tongue | Barmen | Soldiers<br>University men<br>Tobacconists<br>Dock labourers | Agricultural lab. | Farmers. |
| Mouth | Barmen<br>Commer. travellers | Shoemakers<br>Office-staff<br>Hall porters | .. | .. |
| Pharynx | Barmen<br>University men | Office staffs<br>Car men | .. | .. |
| Liver | Weavers | Barmen<br>Teachers<br>Soldiers<br>Fishermen<br>Plumbers<br>Greengrocers | Smiths | .. |
| Pancreas | .. | Barmen<br>Plumbers<br>Commer. travellers | Farmers<br>Agricultural lab. | .. |
| Peritoneum | .. | Fishermen | .. | .. |
| Penis | Smiths | Carpenters<br>General labourers<br>Masters<br>Tailors | .. | .. |
| Jaw | Fishermen | Smiths<br>Railwaymen | .. | .. |
| Lip | .. | Farmers<br>Market gardeners<br>Railway men<br>Police | .. | .. |
| Skin | Farmers<br>Agricultural lab. | Bargemen<br>Fishermen | Railwaymen | .. |
| Kidney | .. | Drapers | Agricultural lab. | .. |

Amongst *wives* of those who carry on the different trades, and *women* in female trades, we find excess or deficiency for the following organs:

TABLE XV—(*cont.*).

| *Organs.* | *Increased.* | *Probably increased.* | *Probably reduced.* | *Reduced.* |
|---|---|---|---|---|
| Stomach | Farmers<br>Farmers' labourers | Market gardeners<br>Shoemakers | Railwaymen<br>Bargemen<br>Firemen<br>Hall porters<br>Lady teachers | University men.<br>Office staff. |
| Oesophagus | Farmers' labourers | .. | .. | .. |
| Lung | Nurses | .. | .. | .. |
| Intestines | .. | Office staff | Farmers' labourers | Domestics (females). |
| Rectum | .. | Masons | .. | .. |
| Bladder | .. | .. | Farmers | .. |
| Tongue | Bargemen | .. | .. | .. |
| Liver | Farmers | Navvies | University men<br>Dealers in fuel<br>Domestics (female) | .. |
| Pancreas | .. | Railwaymen | .. | .. |
| Peritoneum | .. | Railwaymen | .. | .. |
| Cervix uteri | Police<br>Butchers<br>Bargemen<br>Navvies | Publicans<br>Painters<br>Firemen<br>Greengrocers<br>Commer. travellers<br>Hall porters | Market gardeners<br>Stock farmers<br>.. | Farmers<br>Farmers' lab. |
| Breast | Teachers<br>Office staff<br>Commer. travellers | Lady teachers<br>Publicans<br>Painters | Weavers<br>Domestics (female) | .. |
| Ovary | Railwaymen<br>Office staff<br>Hall porters<br>University men<br>Needlewomen | Stock farmers<br>Fishermen<br>Nurses | Farmers' labourers<br>Publicans | .. |
| Uterus (unspecified) | .. | .. | Domestics<br>Farmers | .. |
| Corpus uteri | .. | .. | Farmers' labourers<br>Domestics | .. |
| Skin | .. | General labourers | .. | .. |

### CONCLUSIONS.

It would be interesting to consider what conclusions may be drawn from the figures in this paper, but that would take up too much space. Besides, in my opinion conclusions will only be justifiable when we have sufficient international material at our disposal for comparison.

This material will have to consist of the results of research on many, relatively small, areas during a period shortly before the Second World War, with due observance of the necessary precautions. With larger areas racial, geographical and economic differences in the same trade play a prominent part, and a trade may be quite different in one country from another. During and after the war there was too much changing of occupation.

I wish to express my gratitude to Dr. W. F. Wassink of the Antonie van Leeuwenhoekhuis, Amsterdam, for his interest in this work and for his encouragement. Many thanks are also due to Sir Percy Stocks, for all the help he gave me, particularly in connection with the statistics.